# Sex differences in acute delta-9-tetrahydrocannabinol (Δ^9^-THC) response and tolerance as a function of mouse strain

**DOI:** 10.1007/s00213-023-06421-8

**Published:** 2023-07-29

**Authors:** Courtney F. Lulek, Malabika Maulik, Swarup Mitra, Josée Guindon, Daniel J. Morgan, Angela N. Henderson-Redmond

**Affiliations:** 1 Department of Biomedical Sciences, Marshall University, Huntington, WV 25755 USA; 2 Department of Pharmacology and Neuroscience, Texas Tech University Health Sciences Center, Lubbock, TX 79430 USA

**Keywords:** Sex differences, cannabinoids, pain, ∆^9^-THC, tolerance, mouse strain

## Abstract

Cannabinoids are increasingly used to alleviate pain; however, tolerance to their antinociceptive effects, including those of delta-9-tetrahydrocannabinol (Δ^9^-THC), may limit their therapeutic utility. With more women than men using medical cannabis for pain relief, it is crucial to understand how sex influences cannabinoid-mediated antinociception and tolerance. Though studies in rats consistently find females are more sensitive to the acute antinociceptive effects of cannabinoids, our work with mice consistently finds the converse. The present study examined whether our observed sex differences in Δ^9^-THC-induced antinociception and tolerance are consistent across multiple mouse strains or are strain-dependent. Male and female C57BL/6J (B6), DBA/2, AKR, and CBA/J mice were assessed for differences in acute Δ^9^-THC-induced antinociception and hypothermia prior to and following seven days of once-daily Δ^9^-THC administration. Consistent with our previous findings, male B6 mice were more sensitive to the acute antinociceptive effects of Δ^9^-THC than female littermates, an effect which dissipated with age. B6 males had decreased cannabinoid expression in the PAG compared to females. While DBA and CBA female mice showed increased Δ^9^-THC-antinociception compared to male littermates at 30 and 10 mg/kg Δ^9^-THC, respectively, these differences were less pronounced at higher doses, revealing that dose of Δ^9^-THC may also be important. Overall, CBA mice were more sensitive to Δ^9^-THC-induced antinociception while AKR mice were less responsive. These studies highlight the therapeutic potential of Δ^9^-THC in pain management and underscore the importance of considering not only Δ^9^-THC dose as a function of sex, but potentially genetic differences when evaluating their clinical utility.

**Supplementary Information:**

The online version contains supplementary material available at 10.1007/s00213-023-06421-8.

## Introduction

Chronic pain, defined as prolonged pain lasting 3 months or longer, currently affects over 20% of adults in the United States (Yong et al. 2022). While opioids remain the standard treatment for managing chronic non-cancer pain (Boudreau et al. 2009; Campbell et al. 2010), prolonged use of opioids can result in tolerance, opioid use disorder, overdose, and/or death (Kolodny et al. 2015; Vowles et al. 2015). Increasingly, cannabinoid-based therapies, including the use of delta-9-tetrahydrocannabinol (Δ^9^-THC), a primary cannabinoid component of marijuana, have been investigated as viable alternatives for the management of long-term pain (for a review, see Mücke et al. 2018). The antinociceptive effects of Δ^9^-THC are primarily mediated centrally by cannabinoid type-1 (CB_1_R; Matsuda et al. 1990) and peripherally by cannabinoid type-2 (CB_2_R; Munro et al. 1993) receptors. As with other G protein-coupled receptors (GPCRs), β-arrestin-2 (βArr2)-induced desensitization of CB_1_R represents a primary mechanism through which cannabinoid tolerance occurs (Nguyen et al. 2012; Sim et al. 1996). The resulting tolerance to the antinociceptive effects of cannabinoids represents a significantly limitation to their clinical utility.

Clinically, women report a greater incidence and severity of chronic pain compared to men (Dahlhamer et al. 2018; Nahin 2015). Evidence suggests that sex influences multiple cannabinoid-related outcomes, including the prevalence of cannabinoid use disorders (CUDs; Hernandez-Avila et al. 2004; Khan et al. 2013), abuse liability (Cooper and Haney 2014), withdrawal severity (Copersino et al. 2006), and neuronal activity in those with CUDs (Wetherill et al. 2015). Therefore, it is essential that research investigating the impact of cannabinoids in chronic pain include female subjects. Likewise, with more women than men now using medical cannabis for pain relief (Cuttler et al. 2016), it is imperative to gain a better understanding of how sex influences cannabinoid-mediated antinociception and tolerance.

Findings on cannabinoid sex differences from clinical studies are mixed. For example, some studies find that females are more sensitive (Cooper and Haney 2014; Roser et al. 2009; Wardle et al. 2015) while others found females to be less sensitive (Haney 2007; Penetar et al. 2005) than males to the subjective (“high”) effects of cannabinoids, and some reported no differences (Anderson et al. 2010; Cooper and Haney 2016). Other studies have found that females are less sensitive than males to the objective effects of cannabinoids, including tachycardia (Cooper and Haney 2016; Penetar et al. 2005) and analgesia (Cooper and Haney 2016). One way to assess sex differences in cannabinoid-mediated effects is to utilize rodent models. Data utilizing C57BL6/J (B6) mice consistently finds female wild-type mice to be less sensitive to the antinociceptive effects of ∆^9^-THC and CP55,940 than their male littermates (Henderson-Redmond et al. 2022; Lafleur et al. 2018; Piscura et al. 2023b). However, these findings contrast with work in rats that find the converse (Craft et al. 2012; Moore and Weerts 2021; Romero et al. 2002; Tseng and Craft 2001; Wakley et al. 2014b). Of note, both female mice and rats show accelerated tolerance development to the antinociceptive effects of cannabinoids compared to their male littermates (Henderson-Redmond et al. 2021, 2022; Nguyen et al. 2018, 2020; Parks et al. 2020; Wakley et al. 2014b), suggesting that the observed sex differences in antinociception are due to initial cannabinoid exposure and sensitivity.

As our studies have consistently utilized mice on a B6 background, it remains unknown whether the sex differences we observe are consistent across different mouse lines or whether these differences are strain-specific. Therefore, the main purpose of the present study is to assess sex differences in Δ^9^-THC-induced antinociception in other mouse strains. Likewise, we also attempted to identify whether the sex differences we observe in B6 mice persist at an older age and whether they might be due to basal differences in CB_1_R and/or CB_2_R gene expression in regions mediating antinociception, including the periaqueductal grey (PAG) and spinal cord.

## Methods

### Subjects

Subjects included 281 experimentally naïve (age-matched 8–16 or 78 weeks) adult male and female mice from the following strains: AKR/J (AKR; *N*=52; 26/sex), DBA/2 (DBA; *N*=49; 25 male and 24 female), CBA/J mice (CBA; *N*=86; 43/sex), and C57BL/6J (B6; *N*=94; 47/sex). Mouse strains were chosen based on previous work by Kest et al. (1999) examining sex differences in morphine-induced antinociception dose-response curves across these mouse strains. All mice were obtained from Jackson Laboratories: [C57BL/6J (#000664); DBA/2 (#000671); AKR (#000648); CBA/J (#000656)]. B6 and AKR mouse strains were chosen because morphine produced increased antinociception in males compared to females while CBA females showed the opposite. In contrast, there were no sex differences in morphine-induced antinociception in DBA mice as a function of sex. Mice were group housed (3–5/cage) on a 12:12 hour light/dark cycle (lights out at 18:00) with ad libitum access to food and water. Female mice were not monitored for estrus cycle. Mice were weighed daily prior to any administration of drug to ensure proper dosing. Animal care procedures were conducted in accordance with NIH guidelines for the Care and Use of Laboratory Animals ( 2011) and with approval from Marshall University’s Institutional Animal Care and Use Committee (IACUC).

### Drugs/materials

Delta-9-tetrahydrocannabinol (∆^9^-THC) was obtained from the National Institute on Drug Abuse Drug Supply (Bethesda, MD). For all experiments, ∆^9^-THC was dissolved in 0.9% saline, 5% Cremaphor EL, and 5% ethanol (18:1:1 v/v/v) and administered intraperitoneally (IP) in an injection volume of 10 ml/kg, 60 minutes prior to testing. Doses of Δ^9^-THC were selected based on previous data obtained in our lab that resulted in a 70% maximum possible effect (%MPE) in the tail-flick assay in male mice (Henderson-Redmond et al. 2020, 2021). An additional group of mice was treated with vehicle (VEH) alone to serve as a control group. VEH was prepared using 0.9% saline, 5% Cremaphor EL, and 5% ethanol (18:1:1 v/v/v) and given by IP injection of 10 ml/kg 60 minutes prior to testing. RNAse Zap, Buffer RW1, Buffer RPE, diethyl pyrocarbonate water (DEPC H_2_O), Wipeout Buffer, Quantiscript^®^ Reverse Transcriptase (RT), Quantiscript RT Buffer, RT Primer Mix, and Rnase-free water were obtained from Qiagen, Trizol from Thermo Fisher Scientific, and chloroform from Lab Alley. The Primetime Gene Expression Master Mix, rox reference dye, and Taqman primers (CB_1_, CB_2_, and β-actin) are from IdT Technologies.

### Antinociception and hypothermia assessment

To measure antinociception, a Columbus Instruments TF-1 tail-flick analgesia meter (Columbus, OH) was calibrated to an intensity of 5. To avoid potential tissue damage to the tail, the instrument was programmed to a 10 s cut-off time. The latency of the tail-flick withdrawal was measured prior to and 60 minutes after administration of Δ^9^-THC or VEH. Tail-flick measurements were recorded between 2 and 5 times for each time point and/or dose. The recorded measurements were used to calculate the antinociceptive response as a percent of the maximum possible effect (%MPE) using the following equation: %MPE = [(post-drug latency)−(pre-drug latency)]/[pre-determined cut-off time (10 s)−(pre-drug latency)]×100. Hypothermia was assessed by taking each subject’s body temperature using a mouse rectal thermometer (Physiotemp Instruments, Clifton, NJ) prior to and 60 minutes following injection. Recorded values, in °C, were used to calculate the percent change in body temperature (%Δ) = [(post-body temperature)−(pre-body temperature)/(pre-body temperature)]×100.

### Cumulative dose-response tolerance testing

Male and female AKR, DBA, CBA, and B6 mice were tested using a range of escalating cumulative doses of Δ^9^-THC. Mice were given cumulative doses to generate dose-response curves of 0 (VEH only), 1, 3, 10, 30, and 100 mg/kg Δ^9^-THC (prior to tolerance development) and 0 (VEH only), 3, 10, 30, 100, and 130 mg/kg Δ^9^-THC (after tolerance development) to assess Δ^9^-THC-mediated antinociception and hypothermia. As previously described, tail-flick and body temperature measurements were taken prior to and 60 minutes after administration of VEH or each cumulative dose of Δ^9^-THC. To achieve cumulative dosing, one hour after injection with 1 mg/kg Δ^9^-THC, mice were dosed with 2 mg/kg Δ^9^-THC to generate a cumulative dose of 3 mg/kg and so on for subsequent cumulative doses. Tail-flick antinociception was calculated as %MPE, and body temperature was calculated as %ΔBT. To determine whether repeated administration of once-daily Δ^9^-THC or VEH shifted the initial (pre chronic dosing) dose-response curve, mice were injected with either 30 mg/kg of Δ^9^-THC or an equal amount of VEH (18:1:1) once-daily for seven consecutive days. On the day immediately following the last day of once-daily injections (day 8), full (post chronic dosing) dose-response curves were generated to assess tolerance to the antinociceptive and hypothermic effects of Δ^9^-THC.

### Cannabinoid receptor gene expression

#### Total RNA extraction and cDNA synthesis

To determine whether there were sex differences in CB_1_R or CB_2_R gene expression, 20 naïve B6 mice (10/sex) and 14 naïve CBA mice (7/sex) were sacrificed and the whole brains and spinal cords were dissected. Following dissection, the PAG, hippocampus, and cerebellum of each brain were punched out using the Kent Scientific Adult Mouse Brain Matrix. The isolated brain regions were stored in a −80°C freezer until further use. Total RNA from brain tissue homogenates was extracted using Phenol Chloroform isolation and purified using RNeasy Mini Kit (Qiagen, Germantown, MD). Extracted RNA samples were measured for total RNA concentration and purity using a NanoDrop™ 2000/2000c Spectrophotometer. Subsequently, 100–500 ng of the total RNA (depending on the RNA concentration of the brain region) was converted to complementary DNA (cDNA) using a QuantiTect Reverse Transcription Kit (Qiagen). Each cDNA template reaction was diluted 10-fold using RNase-free water and stored at −20° C until use. Each qPCR assay was performed using 50 ng of cDNA template using Primetime Gene Expression QPCR Master Mix (IDT Technologies, Coralville, IA) with ROX reference dye on Applied Biosystems Step One plus PCR Machine. The primer probe sets used were predesigned PrimeTime qPCR Assays (IDT), as given below:

1. Cnr1 (GenBank® Accession No. NM_007726; 25 bp): GCAAATTTCCTTGTAGCAGAGAG (forward) and TGAGAAAGAGGTGCCAGGA (reverse) and /56FAM/ACAGGTGCC/ZEN/GAGGGAGCTTC/3IABkFQ/ (probe)

2. Cnr2 (GenBank® Accession No XX): GCTTTGGCTTCTTCTACTGGAG (forward) and GCTCTTGGGACCTACGTG (reverse) and /56-FAM/CCCCAGGGT/ZEN/CTTGTGGAGCC/3IABkFQ/ (probe)

3. β-Actin housekeeping gene (GenBank Accession No. NM_007393; 25 bp): GATTACTGCTCTGGCTCCTAG (forward) and GACTCATCGTACTCCTGCTTG (reverse) and /56-FAM/CTGGCCTCA/ZEN/CTGTCCACCTTCC/ 3IABkFQ/(probe)

#### Real-time quantitative PCR (qPCR)

The thermal cycling conditions for qPCR analysis were activated at 95° C for 3 minutes followed by 40 cycles of denaturation at 95°C for 15 seconds and annealing/elongation at 60°C for 1 minute. The data collected from qPCR was analyzed via the ΔΔCt method. This method was derived from the steps described in an article by Livak and Schmittgen (2001). β-Actin was used as an internal control to normalize gene expression. Two-tailed *t*-tests were conducted to determine significant sex-differences in gene expression. Relative mRNA expression of the Cnr1 and Cnr2 genes was determined and normalized to the β-actin reference gene. The fold change expression of the genes was plotted using male wild-type as the calibrator control for further statistical analysis.

### Data analyses

Sample sizes appropriate for each type of experiment were estimated based on power analysis and/or previously published experiments (Morgan et al. 2014). Male and female mice of each strain were randomly assigned to receive vehicle or ∆^9^-THC. Data for the dose-response shifts were analyzed using SPSS version 25.0 (IBM SPSS Statistics, Armonk, New York) to enable 3-way analyses of variance (ANOVAs) while all other data were analyzed using Prism GraphPad (7.05; GraphPad, La Jolla, CA). The median effective dose (ED_50_) for antinociception and hypothermia as well as the 95% confidence intervals (CIs) was determined from initial and post-dose-response curves using nonlinear regression analyses. Differences between ED_50_ values were determined to be significant if the confidence intervals did not overlap. Two- and three-way ANOVAs were run where appropriate with day/dose, sex, and/or time point as the main factors. Because different doses of ∆^9^-THC were used for the pre- (0–100) and post (0–130) dose-response curves, initial two-way ANOVAs assessing mice for sex differences in ∆^9^-THC-mediated sensitivity were done using all doses between 0 and 100 mg/kg while three-way ANVOAs only assessed the common doses used (0, 3, 10, 30, and 100 mg/kg) in the pre- and post-dose-response curves. All doses were used for each curve in calculating ED_50_s. When comparing across lines, only the pre-dose-response curves were used and all doses were included. For all repeated measure analyses done with SPSS, Mauchly’s test of sphericity was calculated to assess equal variances. Where sphericity was violated, the Greenhouse-Geisser correction was used to reduce the probability of making a type I error. When the Greenhouse-Geisser correction is used in reporting degrees of freedom, it has been rounded off to the nearest whole number. Bonferroni post-hoc analyses were performed when significant interaction effects were detected. All data described above are expressed as the mean ± the standard error of the mean (SEM). For all analyses, significance was set at *p*<0.05.

## Results

### Tolerance to ∆9-THC in B6 mice

#### Antinociception

**Fig. 1:**
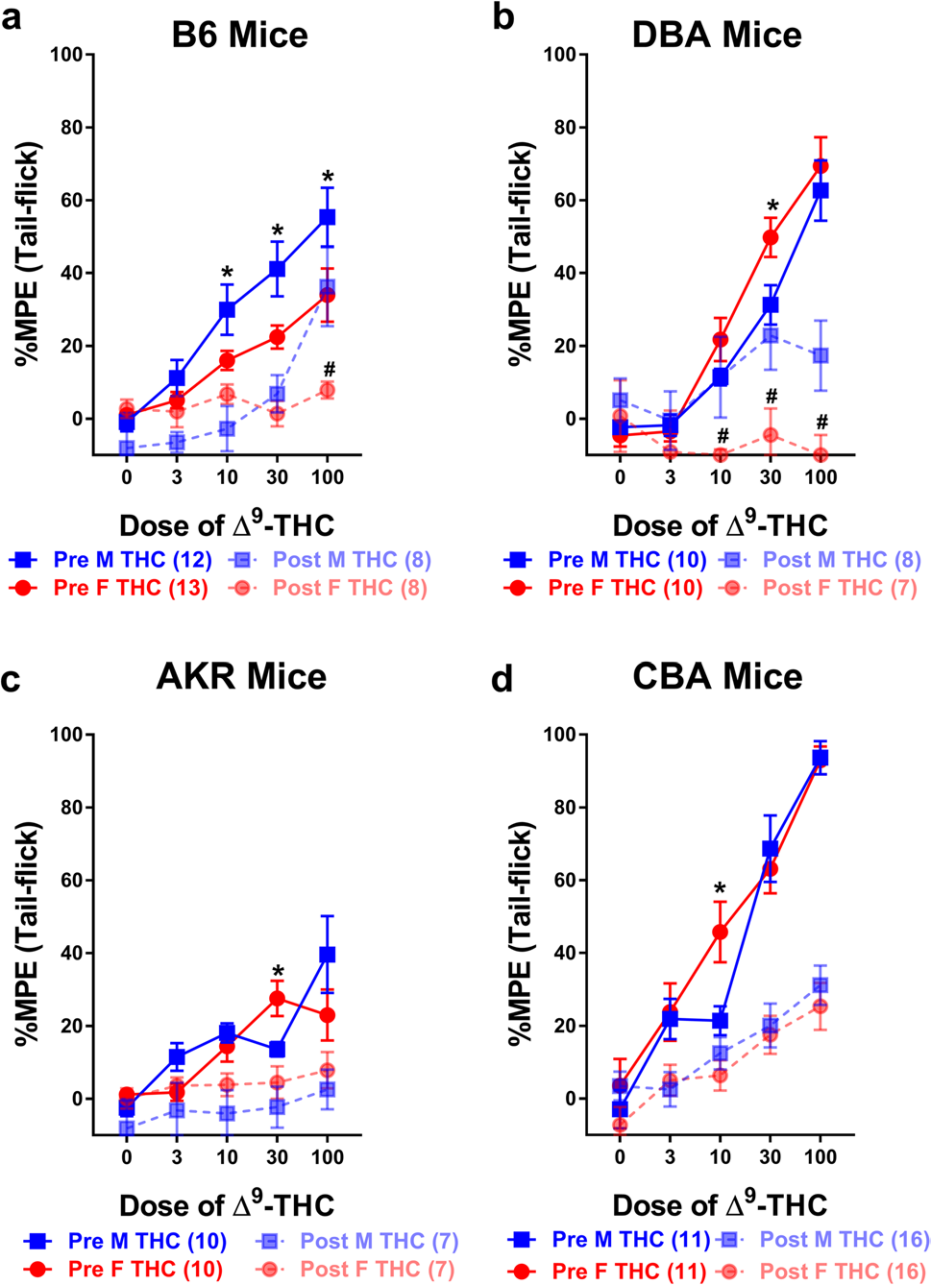
Tolerance development to the antinociceptive effects of ∆^9^-THC assessed via shifts in dose-response curves. Tolerance development to the antinociceptive effects (%MPE) of ∆^9^-THC in both male (blue squares) and female (red circles) **a** B6, **b** DBA, **c** AKR, and **d** CBA mice prior to- (solid lines) and following 7 days (dashed lines) of once-daily treatment with 30 mg/kg ∆^9^-THC. Error bars represent the mean ± SEM; data were analyzed using a three-way ANOVA with Bonferroni post-hoc tests (*p<0.05 comparing male to females within dose in the pre-dose-response; ^#^*p<*0.05 comparing males to females within dose following tolerance development). Sample sizes for each group are in parentheses

Sex differences and tolerance to the antinociceptive effects of ∆^9^-THC were assessed in male and female B6 mice. Results from two-way ANOVAs assessing the initial (pre) dose-response curves revealed main effects of both sex (*F*_1,23_=5.052, *p*=0.034) and dose (*F*_2,55_=40.226, *p*<0.001) though only a trend towards a dose-by-sex interaction (*F*_2,55_=2.706, *p*=0.066). Post-hoc analyses revealed that ∆^9^-THC dose-dependently increased tail-flick antinociception and that males were, overall, more sensitive to the initial antinociceptive effects of ∆^9^-THC (23.373 + 3.270) than female (13.180 + 3.142) B6 mice (Fig. 1a). Results from a three-way ANOVA assessing the development of tolerance following once-daily administration of 30 mg/kg ∆^9^-THC for 7 days followed by the generation of a post-dose-response curve again revealed main effects of dose (*F*_3,96_=37.563, *p*<0.001) and time (*F*_1,37_=21.011, *p*<0.001) and dose-by-sex (*F*_3,96_=7.708, *p*<0.001) and dose-by-time (*F*_3,96_=5.131, *p*=0.004) but not a sex-by-time (*p*=0.157) or a dose-by-time-by-sex (*p*=0.208) interaction. Post-hoc analyses revealed that, collectively, tolerance developed to the antinociceptive effects of ∆^9^-THC in both male (*F*_1,37_=17.884, *p*<0.001) and female (*F*_1,37_=5.009, *p*=0.031) B6 mice. While there was a difference in antinociceptive response between male and female B6 mice prior to tolerance development (*p*=0.016), driven primarily by the effects of ∆^9^-THC at 10, 30, and 100 mg/kg, following tolerance, there was no difference in their overall antinociceptive response (*p*=0.860) barring the increased response males showed at 100 mg/kg (Fig. 1a). Male and female B6 mice administered VEH at all doses tested did not show a significant difference in antinociception either as a function of dose or sex but did differ from mice getting increasing doses of ∆^9^-THC both simultaneously during the pre-dose-response and from mice that received ∆^9^-THC during the post-dose-response following once-daily administration of VEH for 7 days (see Supplemental Table 1A).

#### Hypothermia

**Fig. 2:**
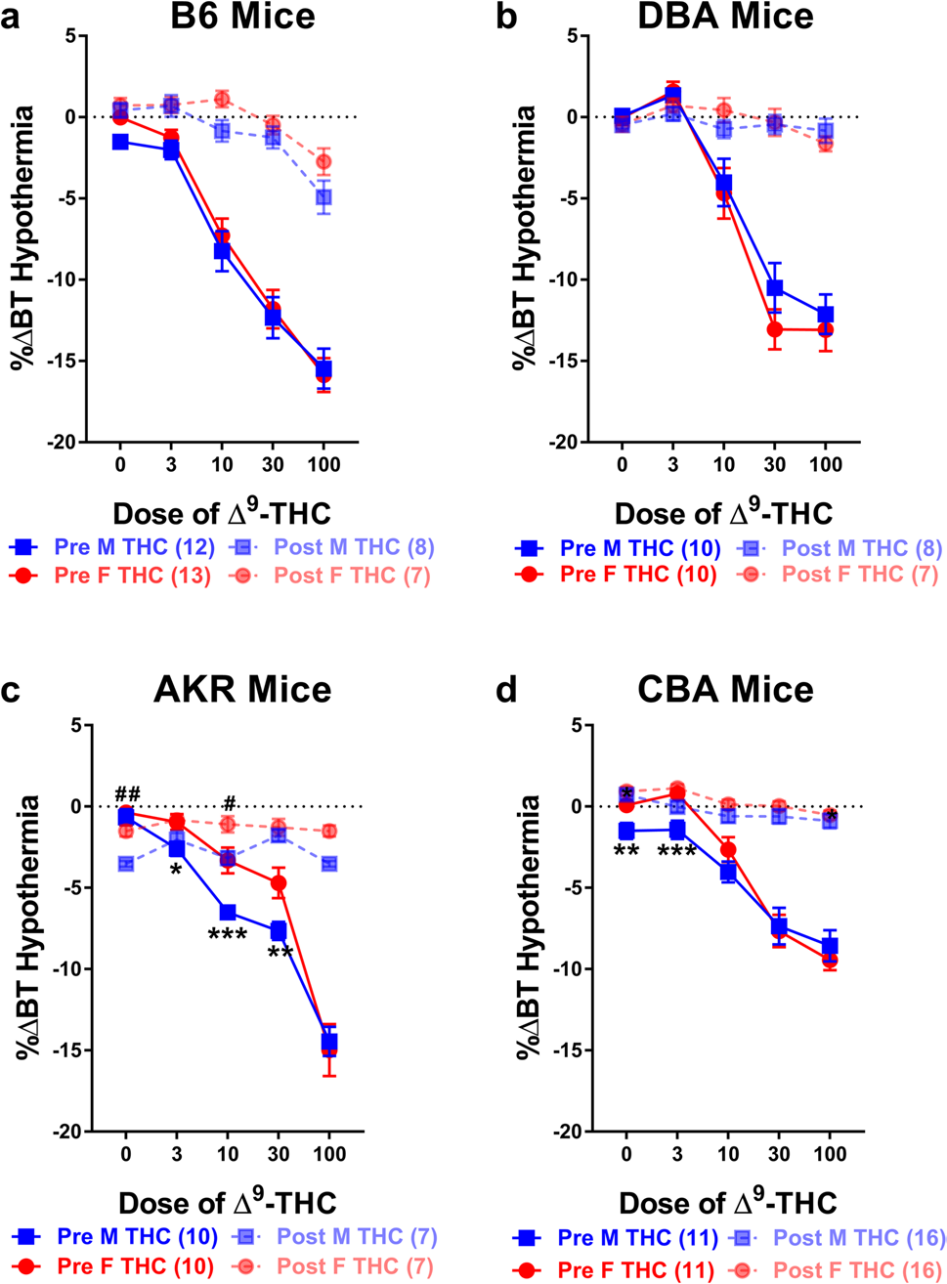
Tolerance development to the hypothermic effects of ∆^9^-THC assessed via shifts in dose-response curves. Tolerance development to the hypothermic effects (%∆BT) of ∆^9^-THC in both male (blue squares) and female (red circles) **a** B6, **b** DBA, **c** AKR, and **d** CBA mice prior to- (solid lines) and following 7 days (dashed lines) of once-daily treatment with 30 mg/kg ∆^9^-THC. Error bars represent the mean ± SEM; data were analyzed using a three-way ANOVA with Bonferroni post-hoc tests (*p<0.05, **p<0.01; ***p<0.001 comparing males to females within dose in the pre-dose-response; ^#^*p<*0.05; ^##^*p*<0.01 comparing males to females within dose following tolerance development). Sample sizes for each group are in parentheses

Sex differences and tolerance to the hypothermic effects of ∆^9^-THC were assessed in male and female B6 mice. Results from two-way ANOVAs assessing the initial (pre) dose-response curves revealed main effects of dose (*F*_2,48_=175.101, *p*<0.001) but not of sex (*p*=0.446) or a dose-by-sex interaction (*p*=0.678). Post-hoc analyses revealed that ∆^9^-THC dose-dependently increased hypothermia and the degree of hypothermia induced was nearly identical in male and female B6 mice (Fig. 2a). Results from a three-way ANOVA assessing the development of tolerance following once-daily administration of 30 mg/kg ∆^9^-THC for 7 days followed by the generation of a post-dose-response curve revealed main effects of dose (*F*_2,89_=135.992, *p*<0.001) and time (*F*_1,37_=106.774, *p*<0.001) and a dose-by-time (*F*_2,89_=48.467, *p*<0.001) interaction. However, there was neither a main effect of sex (*p=*0.206) nor a dose-by-sex (*p*=0.771), sex-by-time (*p*=0.779), or a dose-by-sex-by-time (*p*=0.333) interaction. Post-hoc analyses revealed that, collectively, tolerance developed to the hypothermic effects of ∆^9^-THC in both male (*F*_1,37_=49.726, *p*<0.001) and female (*F*_1,37_=57.246, *p*<0.001) B6 mice and that overall, daily administration of 30 mg/kg decreased the magnitude of hypothermia evoked at all doses tested and that ∆^9^-THC-evoked hypothermia did not differ across dose as a function of sex (Fig. 2a). As with antinociception, male and female B6 mice administered VEH at all doses tested did not show a significant difference in hypothermia either as a function of dose or sex but did differ from mice getting increasing doses of ∆^9^-THC both simultaneously during the pre-dose-response and from mice that received ∆^9^-THC during the post-dose-response following once-daily administration of VEH for 7 days (see Supplemental Table 1b).

### Tolerance to ∆^9^-THC in DBA mice

#### Antinociception

Sex differences and tolerance to the antinociceptive effects of ∆^9^-THC were also assessed in male and female DBA mice. Results from two-way ANOVAs assessing the initial (pre) dose-response curves revealed a main effect of dose (*F*_2,42_=94.622, *p*<0.001) but not of sex (*p*=0.094) or a dose-by-sex interaction (*p*=0.209). Post-hoc analyses revealed that ∆^9^-THC dose-dependently increased tail-flick antinociception and that females trended towards being more sensitive to the initial antinociceptive effects of ∆^9^-THC (50.813 + 2.707) than male (15.818 + 2.707) DBA mice (Fig. 1b). Results from a three-way ANOVA assessing the development of tolerance following once-daily administration of 30 mg/kg ∆^9^-THC for 7 days followed by the generation of a post-dose-response curve again revealed main effects of dose (*F*_4,124_=38.001, *p*<0.001) and time (*F*_1,31_=20.654, *p*<0.001) and a dose-by-time (*F*_4,124_=25.495, *p*<0.001), time-by-sex (*F*_1,31_=6.999, *p*=0.013), and a dose-by-sex-by-time (*F*_4,124_=3.241, *p*=0.014) interaction. In contrast, there was neither a main effect of sex (*p*=0.200) nor a dose-by-sex (*p*=0.882) interaction. Post-hoc analyses revealed that female DBA mice were more sensitive to the antinociceptive effects of 30 mg/kg ∆^9^-THC than male DBA mice in the pre-dose-response curve (*p*=0.047). Interestingly, following 7 days of once-daily treatment with 30 mg/kg ∆^9^-THC, female DBA mice showed evidence of tolerance development to doses of 10, 30, and 100 mg/kg of ∆^9^-THC (all *p*<0.001) while male DBA mice only showed evidence of tolerance following the highest dose of ∆^9^-THC tested (100 mg/kg, *p*<0.001). Likewise, female DBA mice showed evidence of more rapid tolerance development to the antinociceptive effects of ∆^9^-THC as they showed a significantly decreased response compared to male DBA mice following administration of 10 (*p*=0.017), 30 (*p*=0.013), and 100 (*p*=0.035) mg/kg ∆^9^-THC (Fig. 1b). Male and female DBA mice administered VEH at all doses tested did not show a significant difference in antinociception either as a function of dose or sex but did differ from mice getting increasing doses of ∆^9^-THC both simultaneously during the pre-dose-response and from mice that received ∆^9^-THC during the post-dose-response following once-daily administration of VEH for 7 days (see Supplemental Table 2A).

#### Hypothermia

Sex differences and tolerance to the hypothermic effects of ∆^9^-THC were assessed in male and female DBA mice. Results from two-way ANOVAs assessing the initial (pre) dose-response curves revealed a main effect of dose (*F*_3,47_=101.663, *p*<0.001) but not of sex (*p*=0.559) or a dose-by-sex interaction (*p*=0.469). Post-hoc analyses revealed ∆^9^-THC dose-dependently increased hypothermia equally across both male (−4.025 + 0.629) and female (−4.683 + 0.629) DBA mice (Fig. 2b). Results from a three-way ANOVA assessing the development of tolerance following once-daily administration of 30 mg/kg ∆^9^-THC for 7 days followed by the generation of a post-dose-response curve revealed main effects of dose (*F*_3,85_=70.549, *p*<0.001) and time (*F*_1,31_=62.665, *p*<0.001) and a dose-by-time (*F*_3,85_=53.574, *p*<0.001) interaction. There was neither a main effect of sex (*p*=0.641) or a dose-by-sex (*p*=0.532), sex-by-time (*p*=0.433), or a sex-by-time-by-dose (*p*=0.672) interaction. Post-hoc analyses revealed that, collectively, tolerance developed to the hypothermic effects of ∆^9^-THC in both male (*F*_1,31_=26.362, *p*<0.001) and female (*F*_1,31_=36.546, *p*<0.001) DBA mice. Post-hoc results also revealed that following once-daily administration of 30 mg/kg ∆^9^-THC, the severity of hypothermia evoked following administration of 10 (*p*=0.003), 30 (*p*<0.001), and 100 (*p*<0.001) mg/kg ∆^9^-THC was significantly decreased in the post-dose-response curve compared to the pre-dose-response curve for both male and female DBA mice (Fig. 2b). As with antinociception, male and female DBA mice administered VEH at all doses tested did not show a significant difference in hypothermia either as a function of dose or sex but did differ from mice getting increasing doses of ∆^9^-THC both simultaneously during the pre-doseresponse and from mice that received ∆^9^-THC during the post-dose-response following once-daily administration of VEH for 7 days (see Supplemental Table 2B).

### Tolerance to ∆^9^-THC in AKR mice

#### Antinociception

Sex differences and tolerance to the antinociceptive effects of ∆^9^-THC were assessed in male and female AKR mice. Results from two-way ANOVAs assessing the initial (pre) dose-response curves revealed a main effect of dose (*F*_2,35_=14.435, *p*<0.001) but neither a main effect of sex (*p*=0.070) nor a dose-by-sex interaction (*p*=0.310). Post-hoc analyses revealed that ∆^9^-THC at higher doses (10–100 mg/kg) marginally increased tail-flick antinociception compared to lower doses. While there was no significant sex difference in ∆^9^-THC induced antinociception, male AKR mice trended towards an overall increase in ∆^9^-THC-induced antinociception (14.515 + 2.163) compared to female (11.320 + 2.163) AKR littermates (Fig. 1c). Results from a three-way ANOVA assessing the development of tolerance following once-daily administration of 30 mg/kg ∆^9^-THC for 7 days followed by the generation of a post-dose-response curve revealed main effects of dose (*F*_2,67_=12.004, *p*<0.001) and time (*F*_1,30_=18.517, *p*<0.001) and a dose-by-time (*F*_2,67_=4.378, *p*=0.013), but not a dose-by-sex (*p*=0.146), sex-by-time (*p*=0.167), or a dose-by-time-by-sex (*p*=0.209) interaction. Post-hoc analyses revealed that tolerance developed to the antinociceptive effects of ∆^9^-THC in both male (*F*_1,30_=16.357, *p*<0.001) and female (*F*_1,30_=4.167, *p*=0.050) AKR mice. While there were no differences in any doses as a function of sex, the development of tolerance was driven by ∆^9^-THC-induced antinociception at 10, 30, and 100 mg/kg in the pre-dose-response curve (14.819 + 2.140) as ∆^9^-THC failed to induce an antinociceptive response that differed from 0 (0.469 + 2.558) in the post-dose-response curve following tolerance development (Fig. 1c). Male and female AKR mice administered VEH at all doses tested did not show a significant difference in antinociception either a function of dose or sex from each other. Given the low antinociceptive response, female mice getting VEH differed only from female mice getting increasing doses of ∆^9^-THC simultaneously during the pre-dose response. Interestingly, VEH-treated mice did not differ from mice that received ∆^9^-THC during the post-dose response following once-daily administration of VEH for 7 days, likely due to the lack of a robust antinociceptive response in AKR mice to ∆^9^-THC (see Supplemental Table 3A).

#### Hypothermia

Sex differences and tolerance to the hypothermic effects of ∆^9^-THC were assessed in male and female AKR mice. Results from two-way ANOVAs assessing the initial (pre) dose-response curves revealed a main effect of dose (*F*_2,38_=162.580, *p*<0.001) but only trends for a main effect of sex (*p*=0.055) and a dose-by-sex interaction (*p*=0.053). Post-hoc analyses revealed that ∆^9^-THC dose-dependently increased hypothermia and the degree of hypothermia induced trended towards being more severe in male (−5.562 ± 0.490) versus female (−4.142 ± 0.490) AKR mice (Fig. 2c). Results from a three-way ANOVA assessing the development of tolerance following once-daily administration of 30 mg/kg ∆^9^-THC for 7 days followed by the generation of a post-dose-response curve again revealed a main effects of dose (*F*_2,66_=106.802, *p*<0.001), sex (*F*_1,30_=9.557, *p*=0.004), and time (*F*_1,30_=52.250, *p*<0.001) as well as a dose-by-time (*F*_2,66_=94.358, *p*<0.001) and a dose-by-sex-by-time (*F*_2,66_=3.396, *p*=0.035) interaction. In contrast, there was neither a dose-by-sex (*p*=0.201) or a sex-by-time (*p*=0.956) interaction. Post-hoc analyses revealed that, collectively, tolerance developed to the hypothermic effects of ∆^9^-THC in both male (*F*_1,30_=25.729, *p*<0.001) and female (*F*_1,30_=26.524, *p*<0.001) AKR mice. Post-hoc results also revealed that overall, daily administration of 30 mg/kg ∆^9^-THC decreased the post-dose-response effects of 10, 30, and 100 (all *p*<0.001) mg/kg ∆^9^-THC with male AKR mice being more sensitive to the hypothermic effects of ∆^9^-THC both prior to (*p*=0.025) and following (*p*=0.049) ∆^9^-THC tolerance compared to female AKR mice (Fig. 2c). As with antinociception, male and female AKR mice administered VEH at all doses tested did not show a significant difference in hypothermia either a function of dose or sex but did differ from mice getting increasing doses of ∆^9^-THC simultaneously during the pre-dose-response. Female (but not male) AKR mice receiving VEH in the pre-dose-response curve differed from female mice that received ∆^9^-THC during the post-dose-response following once-daily administration of VEH for 7 days (see Supplemental Table 3B).

### Tolerance to ∆^9^-THC in CBA mice

#### Antinociception

Sex differences and tolerance to the antinociceptive effects of ∆^9^-THC were assessed in male and female CBA mice. Results from two-way ANOVAs assessing the initial (pre) dose-response curves revealed a main effect of dose (*F*_5,100_=82.700, *p*<0.001) but neither a main effect of sex (*p*=0.365) or a dose-by-sex interaction (*p*=0.158). Post-hoc analyses revealed that ∆^9^-THC dose-dependently increased tail-flick antinociception. There was no significant sex difference in ∆^9^-THC induced antinociception since male CBA mice (34.977 + 3.680) and female CBA (39.806 + 3.680) littermates displayed equivalent antinociception (Fig. 1d). Results from a three-way ANOVA assessing the development of tolerance following once-daily administration of 30 mg/kg ∆^9^-THC for 7 days followed by the generation of a post-dose-response curve revealed main effects of dose (*F*_4,200_=115.488, *p*<0.001) and time (*F*_1,50_=60.439, *p*<0.001) and a dose-by-time (*F*_4,200_=29.704, *p*<0.001) and a trend towards a dose-by-sex-by-time (*p*=0.054) interaction. In contrast, there was not a main effect of sex (*p*=0.926), a dose-by-sex (*p*=0.218), or a sex-by-time (*p*=0.232) interaction. Post-hoc analyses revealed that tolerance developed to the antinociceptive effects of ∆^9^-THC in both male (*F*_1,50_=21.538, *p*<0.001) and female (*F*_1,50_=40.368, *p*<0.001) CBA mice. While there were no overall sex differences in ∆^9^-THC-induced antinociception between male (27.224 + 2.867) and female (27.604 + 2.867) CBA mice, female mice showed a much greater antinociceptive response following administration of 10 mg/kg ∆^9^-THC in the pre-dose-response (45.771 + 5.742) than male CBA mice (21.408 + 5.742). In contrast, following tolerance, there were no sex differences between males and females in antinociceptive response (Fig. 1d). Male and female CBA mice administered VEH at all doses tested did not show a significant difference in antinociception either as a function of dose or sex but did differ from mice getting increasing doses of ∆^9^-THC both simultaneously during the pre-dose-response and from mice that received ∆^9^-THC during the post-dose-response following once-daily administration of VEH for 7 days (see Supplemental Table 4A).

#### Hypothermia

Sex differences and tolerance to the hypothermic effects of ∆^9^-THC were assessed in male and female CBA mice. Results from two-way ANOVAs assessing the initial (pre) dose-response curves revealed a main effect of dose (*F*_3,56_=84.095, *p*<0.001) but neither a main effect of sex (*p*=0.107) or a dose-by-sex interaction (*p*=0.120). Post-hoc analyses revealed that ∆^9^-THC dose-dependently increased hypothermia to a similar degree in male and female CBA mice (Fig. 2d). Results from a three-way ANOVA assessing the development of tolerance following once-daily administration of 30 mg/kg ∆^9^-THC for 7 days followed by the generation of a post-dose-response curve again revealed main effects of dose (*F*_3,141_=115.338, *p*<0.001), sex (*F*_1,50_=4.287, *p*=0.044), and time (*F*_1,50_=150.434, *p*<0.001) as well as a dose-by-time (*F*_3,141_=63.740, *p*<0.001) and a dose-by-sex (*F*_3,141_=3.271, *p*=0.026) interaction. There was not a dose-by-time (*p*=0.786) or a sex-by-time-by-dose (*p*=0.119) interaction. Post-hoc analyses revealed that tolerance developed to the hypothermic effects of ∆^9^-THC in both male (*F*_1,50_=78.606, *p*<0.001) and female (*F*_1,50_=71.903, *p*<0.001) CBA mice with female CBA mice showing a greater degree of overall ∆^9^-THC-induced hypothermia (−2.423 + 0.242) compared to male (−1.713 + 0.242) CBA mice. Post-hoc results also revealed that overall, daily administration of 30 mg/kg ∆^9^-THC decreased hypothermia evoked in the post-dose-response curve following administration of all doses (0-100 mg/kg) in male CBA mice and following administration of 10, 30, and 100 mg/kg of ∆^9^-THC in female CBA mice (Fig. 2d). As with antinociception, male and female CBA mice administered VEH at all doses tested did not show a significant difference in hypothermia either as a function of dose or sex but did differ from mice getting increasing doses of ∆^9^-THC both simultaneously during the pre-dose-response and from mice that received ∆^9^-THC during the post-dose-response following once-daily administration of VEH for 7 days (see Supplemental Table 4B).

### Strain differences in ED_50_ shifts following ∆^9^-THC tolerance

To assess the development of tolerance to ∆^9^-THC-induced antinociception and hypothermia, the ED_50_ values for the initial (pre) dose-response and following once-daily ∆^9^-THC administration (post-dose-response) curves were calculated for both sexes and for each mouse strain assessed (B6, DBA, AKR, and CBA). While the generation of pre-dose-response curves assessing ∆^9^-THC-induced antinociception (Table 1) and hypothermia (Table 2) enabled the calculation of ED_50_ values, the near complete development of tolerance to ∆^9^-THC-induced antinociception and hypothermia made it difficult to determine ED_50_ values for the post-dose-response curves often resulting in undefined values and/or confidence intervals. However, the pre-dose-response values reveal a much greater variation in ∆^9^-THC-induced antinociception across strain than for ∆^9^-THC-induced hypothermia.

**Table 1:** Calculated ED_50_ values (mg/kg) assessing the antinociceptive effects of Δ^9^-THC across mouse strain

Drug	Genotype	Sex	Pre-drug ED_50_ (CI)	Post-drug ED_50_ (CI)
Δ^9^-THC Tail-flick	B6 mouse	Male	59.37 (36.74–118.9)	187.1 (132.4–458.9)*
		Female	298.4 (160–934.3)^**#**^	6168 (160.9–ND)
	DBA mouse	Male	62.01 (49.14–80.65)	4077 (253–ND)*
		Female	36.8 (27.82–49.92)	ND
	AKR mouse	Male	342.1 (132.9–4639)	373 (ND–ND)
		Female	778.5 (251.8–13204)	104762 (290.2–ND)
	CBA mouse	Male	18.22 (13.69–23.80)	81.3 (54.47–137.5)*
		Female	12.42 (8.446–18.21)	271.9 (135–1725)*

ED_50_ values were calculated from initial dose-response curves generated using non-linear regression analysis. Values shown are mean ED_50_ dose and 95% confidence intervals (CI) for Δ^9^-THC in male and female B6, DBA, AKR, and CBA mice. Significance was determined if the confidence intervals did not overlap. In cases where confidence intervals were undefined, we could not determine if the confidence intervals overlapped, and shifts were unable to be deemed significant

*ND* unable to define the curve or 95% confidence interval

*A significant difference in ED_50_ between pre- and post-dose-response curves

^#^A significant difference between male and females

**Table 2:** Calculated ED_50_ values (mg/kg) assessing the hypothermic effects of Δ^9^-THC across mouse strain

Drug	Genotype	Sex	Pre-drug ED_50_ (CI)	Post-drug ED_50_ (CI)
Δ^9^-THC Hypothermia	B6 mouse	Male	10.80 (8.191–19.01)	29.68 (8.274–ND)
		Female	12.49 (9.552–16.33)	34.04 (20.03–ND)*
	DBA mouse	Male	12.33 (9.128–ND)	ND
		Female	10.13 (ND–ND)	58.91 (18.67–ND)
	AKR mouse	Male	18.18 (13.16–25.47)	95.43 (ND–ND)
		Female	39.31 (10.84–ND)	ND
	CBA mouse	Male	12.98 (ND–20.04)	11.04 (ND–ND)
		Female	14.43 (10.84–ND)	20.06 (ND–53.2)

ED_50_ values were calculated from initial dose-response curves generated using non-linear regression analysis. Values shown are mean ED_50_ dose and 95% confidence intervals (CIs) for Δ^9^-THC in male and female B6, DBA, AKR, and CBA mice. Significance was determined if the confidence intervals did not overlap. In cases where confidence intervals were undefined, we could not determine if the confidence intervals overlapped, and shifts were unable to be deemed significant

*ND* unable to define the curve or 95% confidence interval

*A significant difference in ED_50_ between pre- and post-dose-response curves

### Differences in ∆^9^-THC sensitivity across mouse line

#### Antinociception

**Fig. 3:**
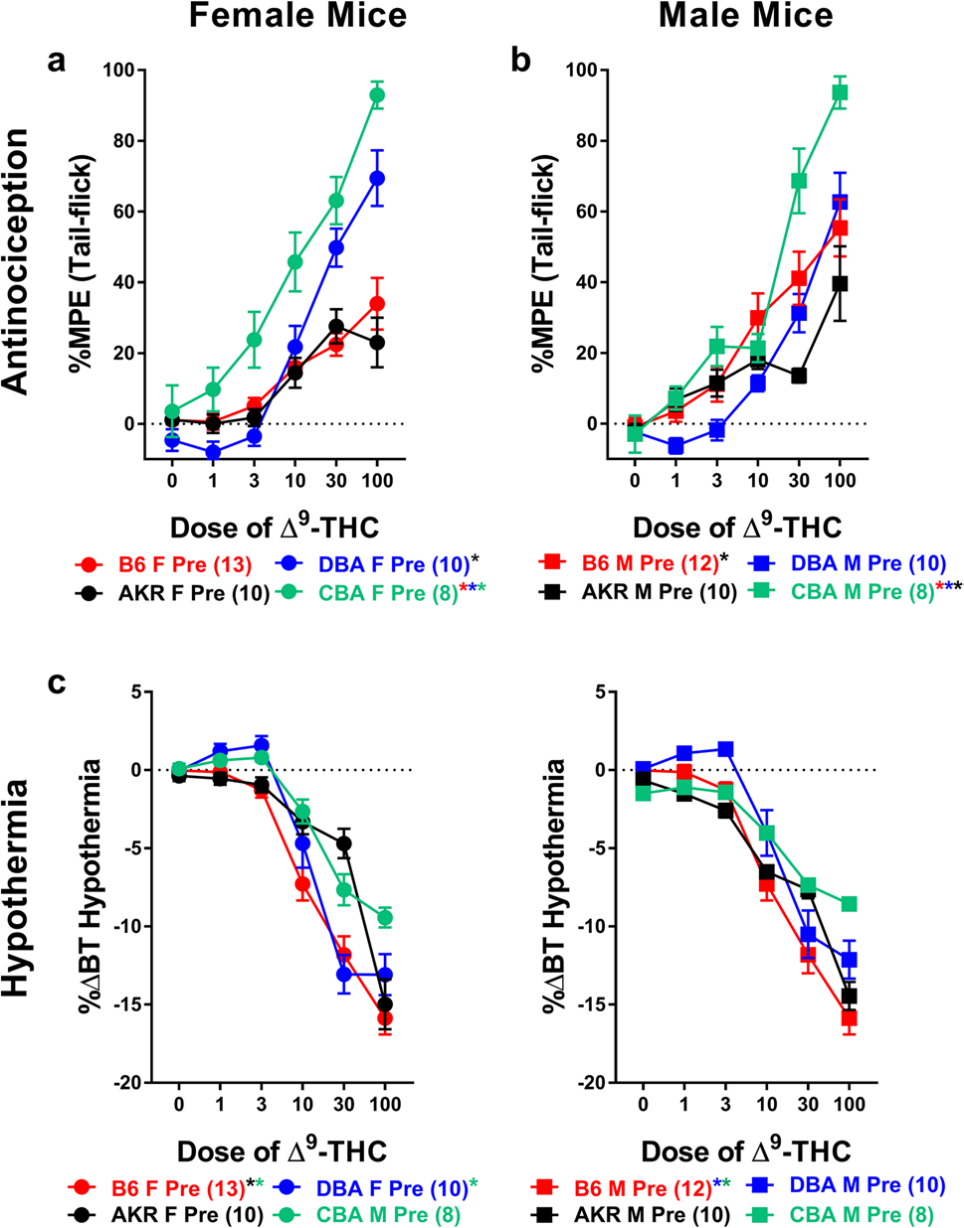
∆^9^-THC-induced antinociception results in greater variability across mouse strain than ∆^9^-THC-induced hypothermia. Assessment of ∆^9^-THC-induced antinociception (%MPE; top) and hypothermia (%∆BT; bottom) in female (circles, **a** and **c**) and male (squares, **b** and **d**) B6 (red), DBA (blue), AKR (black), and CBA (green) mice. Error bars represent the mean ± SEM; data were analyzed using a three-way ANOVA with Bonferroni post-hoc tests (*p<0.05 comparing line to the mouse strain denoted by the same color). Sample sizes for each group are in parentheses

To better understand the role that strain plays in mediating differences in ∆^9^-THC-induced antinociception, male and female B6, DBA, AKR, and CBA mice were assessed across their initial (pre) dose-response curves for differences in sensitivity to the antinociceptive effects of ∆^9^-THC. The (pre) dose-response curves used for this analysis were the same ones used in Fig. 1, but included all doses tested. Results of a three-way ANOVA (comparing sex, mouse strain, and dose) revealed main effects of dose (*F*_3,265_=207.897, *p*<0.001) and mouse strain (*F*_3,79_=24.332, *p*<0.001) and mouse strain-by-sex (*F*_3,79_=2.981, *p*=0.036), dose-by-mouse strain (*F*_10,265_=11.706, *p*<0.001), and a dose-by-sex-by-mouse strain (*F*_10,265_=2.321, *p*=0.012) interactions. In contrast, there was neither a main effect of sex (*p*=0.684) nor a sex-by-dose (*p*=0.105) interaction. Post-hoc analyses revealed that ∆^9^-THC dose-dependently increased antinociception. Post-hoc analyses also revealed that the main effect of mouse strain is driven by the CBA mice whom, overall, showed greater ∆^9^-THC-induced antinociception (37.391 + 2.158) compared to B6 (18.277 + 2.026; *p*<0.001), DBA (18.316 + 2.263; *p*<0.001), and AKR (12.917 + 2.263; *p*<0.001) mice (Fig. 3a, b). Likewise, the sex-by-mouse strain interaction is due to the sex difference between male (23.373 + 2.922) and female (13.180 + 2.807; *p*=0.014) B6 mice in overall ∆^9^-THC-induced antinociception (no other strain differed as a function of sex). Among CBA mice, both males and females showed greater ∆^9^-THC-induced antinociception compared to all other strains assessed. Further, female DBA mice also showed greater ∆^9^-THC-induced antinociception (20.813 + 3.201) than AKR females (11.320 + 3.201, *p*=0.039; Fig. 3a) while among male mice, B6 male mice showed greater ∆^9^-THC-induced antinociception (23.373 + 2.922) compared to AKR males (14.515 + 3.201, *p*=0.044; Fig. 3b).

#### Hypothermia

To better understand the role that strain plays in mediating differences in ∆^9^-THC-induced hypothermia, male and female B6, DBA, AKR, and CBA mice were assessed across their initial (pre) dose-response curves for differences in sensitivity to the hypothermic effects of ∆^9^-THC. The (pre) dose-response curves used for this analysis were the same ones used in Fig. 2, but included all doses tested. Results of a three-way ANOVA (comparing sex, mouse strain, and dose) revealed main effects of dose (*F*_3,218_=469.598, *p*<0.001) and mouse strain (*F*_3,79_=10.562, *p*<0.001) and a mouse strain-by-dose (*F*_8,218_=12.465, *p*<0.001) interaction. In contrast, there was neither a main effect of sex (*p*=0.130) or a dose-by-sex (*p*=0.096), sex-by-mouse strain (*p*=0.308), or a dose-by-sex-by-mouse strain (*p*=0.475) interaction. Post-hoc analyses revealed that ∆^9^-THC dose-dependently increased hypothermia across all mice (Fig. 3c, d). Post-hoc analyses also revealed that among females, CBA mice (−3.038 + 0.546) showed less ∆^9^-THC-induced hypothermia compared to B6 (−6.064 + 0.502; *p*<0.001) and DBA (−4.683 + 0.573; *p*=0.041) mice and that female AKR mice were less sensitive to ∆^9^-THC-induced hypothermia (−4.142 + 0.573; *p*=0.014) compared to B6 female mice (Fig. 3c). Among male mice, B6 mice (−6.733 + 0.523) showed increased ∆^9^-THC-induced hypothermia compared to both male DBA (−4.025 + 0.573; *p*<0.001) and CBA (−3.995 + 0.546; *p*<0.001) mice (Fig. 3d). Taken together, these data suggest that there is greater variability in ∆^9^-THC-induced antinociception compared to hypothermia as a function of strain. Likewise, where there are sex differences, they tend to manifest more in ∆^9^-THC-induced antinociception versus hypothermia.

### Differences in ∆^9^-THC sensitivity as a function of age

#### Antinociception

**Fig. 4:**
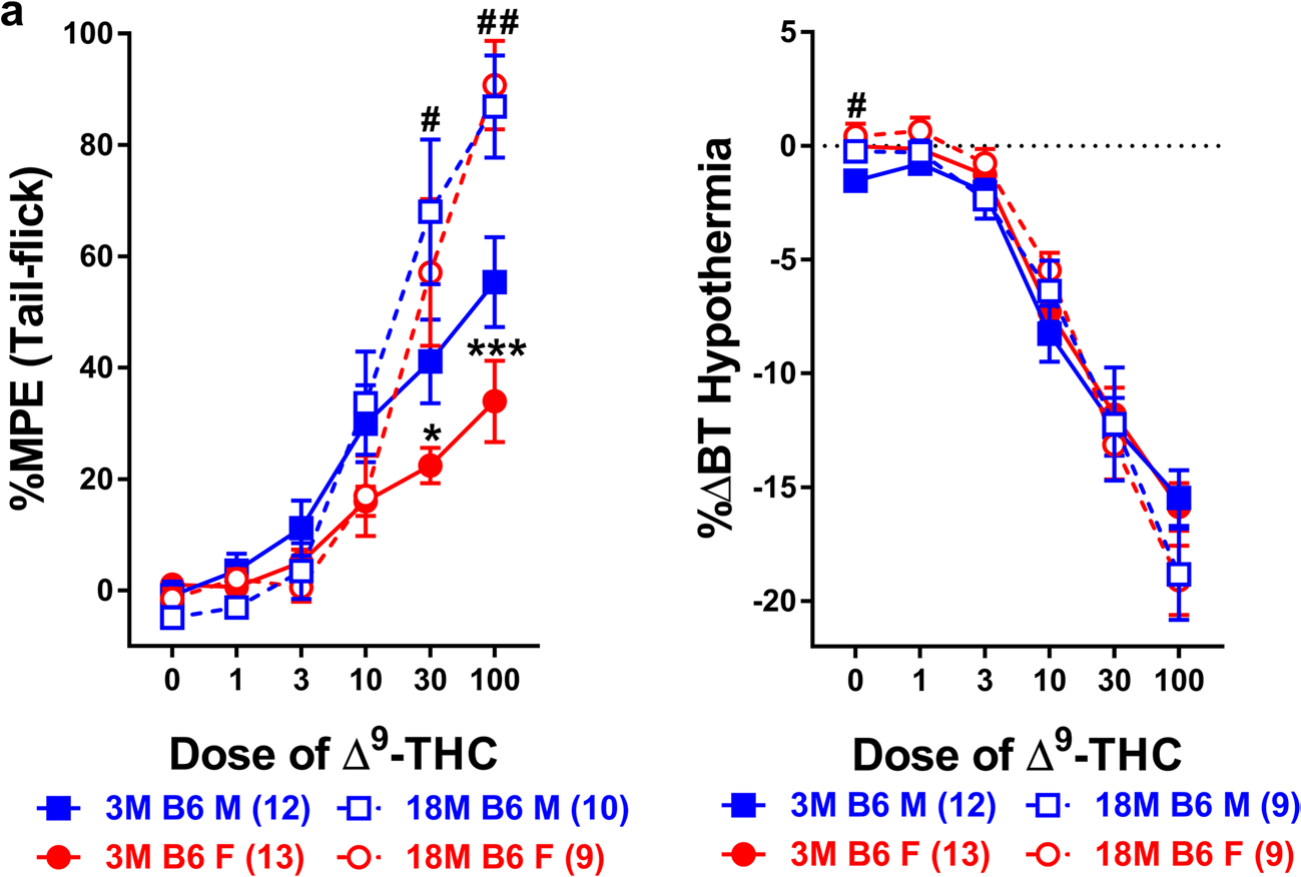
Older B6 mice are more sensitive to the antinociceptive (but not hypothermic) effects of ∆^9^-THC than their younger counterparts. The antinociceptive (%MPE; **a**) and hypothermic (%∆BT; **b**) effects of ∆^9^-THC were assessed in both younger (~8-16 week old; filled symbols) and older (~78-80 week old; unfilled symbols) male (blue squares) and female (red circles) B6 mice. Error bars represent the mean ± SEM; data were analyzed using a three-way ANOVA with Bonferroni post-hoc tests (*p<0.05, ***p<0.001 comparing younger and older females within dose; ^#^*p<*0.05; ^##^*p*<0.01 comparing younger and older males within dose). Sample sizes for each group are in parentheses

To better determine whether age plays a role in mediating differences in ∆^9^-THC-induced antinociception and/or hypothermia, naïve male and female 18-month-old B6 mice were assessed for basal differences across a range of ∆^9^-THC doses to generate initial (pre) dose-response curves for both tail-flick antinociception and hypothermia (Fig. 4). Results from a two-way ANOVA revealed a main effect of dose (*F*_2,42_=65.958, *p*<0.001) but not of sex (*p*=0.640) or a dose-by-sex (*p*=0.431) interaction. Post-hoc analyses revealed that increasing doses of ∆^9^-THC dose-dependently increased antinociception in the tail-flick test regardless of sex (Fig. 4a). Likewise, results from a three-way ANOVA comparing older (18 months) to younger (3 months) B6 mice across a range of ∆^9^-THC (pre) doses revealed main effects of dose (*F*_3,110_=114.886, *p*<0.001) and age (*F*_1,40_=8.334, *p*=0.006) but not of sex (*p*=0.088). Likewise, there was a dose-by-age (*F*_3,110_=16.489, *p*<0.001) but no dose-by-sex (*p*=0.099), sex-by-age (*p*=0.346), or a sex-by-dose-by-age (*p*=0.346) interaction. Post-hoc analyses revealed that ∆^9^-THC dose-dependently increased antinociception in all mice. While there was not a main effect of sex, younger female mice (13.180 + 3.435) were, overall, less sensitive to the antinociceptive effects of ∆^9^-THC than younger male mice (23.373 + 3.575; *p*=0.046) while there was no difference in ∆^9^-THC-induced antinociception between older male (30.669 + 3.916) and female (27.671 + 4.128; *p*=0.601) mice. Compared to younger B6 mice, older B6 (29.170 + 2.845) mice were more sensitive to the effects of ∆^9^-THC than younger (18.277 + 2.479) mice, particularly at doses of 30 and 100 mg/kg ∆^9^-THC (Fig. 4a).

#### Hypothermia

Male and female 18-month-old B6 mice were assessed for basal differences in ∆^9^-THC-induced hypothermia across a range of doses (Fig. 4b). Results from a two-way ANOVA revealed a main effect of dose (*F*_2,28_=128.030, *p*<0.001) but not of sex (*p*=0.707) or a dose-by-sex (*p*=0.629) interaction. Post-hoc analyses revealed that ∆^9^-THC dose-dependently increased antinociception in the tail-flick test regardless of sex (Fig. 4b). Likewise, results from a three-way ANOVA comparing older (18 month) and younger (3 month) B6 mice across a range of ∆^9^-THC (pre) doses, revealed a main effect of dose (*F*_2,80_=301.133, *p*<0.001) and a dose-by-age (*F*_2,80_=4.648, *p*=0.012) interaction. There was not a main effect of sex (*p*=0.440) or age (*p*=0.921) or any dose-by-sex (*p*=0.531), sex-by-age (*p*=0.904), or dose-by-sex-by-age (*p*=0.782) interactions. Post-hoc analyses revealed that ∆^9^-THC dose-dependently induced hypothermia in all mice (females: -6.146 + 0.520; males: -6.725 + 0.528). Neither older female (*p*=0.876) nor male (*p*=0.988) B6 mice differed from their younger counterparts as a function of age. Likewise younger mice did not differ from older mice in ∆^9^-THC-induced hypothermia as a function of sex [older mice (*p*=0.668); younger mice (*p*=0.490); Fig. 4b].

### Differences in gene expression

#### B6 mice

**Fig. 5:**
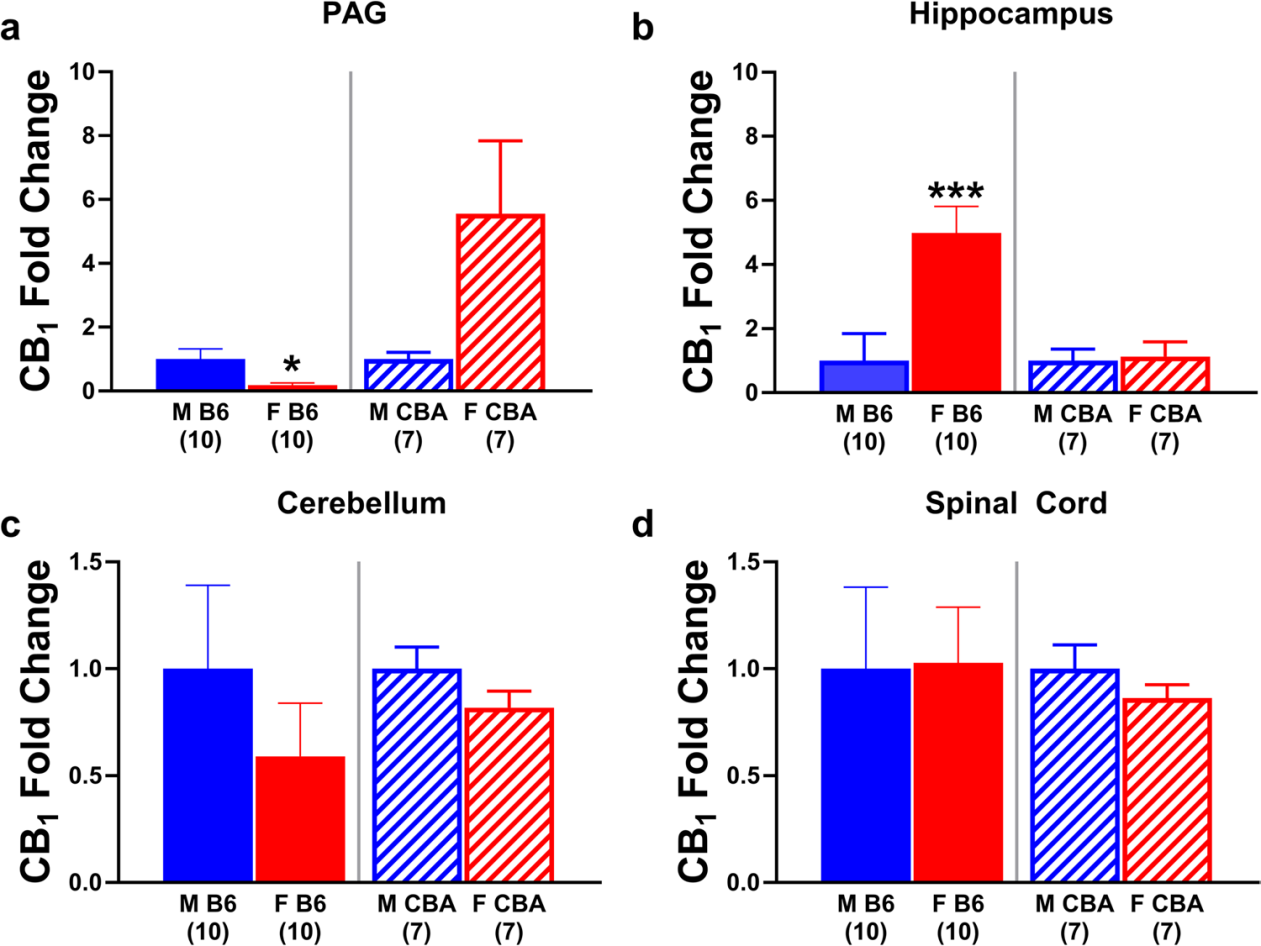
CB_1_R mRNA expression in the PAG of naïve mice parallels observed sex differences in ∆^9^-THC-induced antinociception in B6 and CBA mice. The fold change in CB_1_R expression was determined for naïve male (blue) and female (red) B6 (left panels, solid bars) and CBA (right panels, striped bars) mice in the **a** PAG, **b** hippocampus, **c** cerebellum, and **d** spinal cord. Error bars represent the mean ± SEM; data were analyzed using unpaired two-tailed t-tests (*p<0.05, ***p<0.001 comparing sex within strain). Sample sizes for each group are in parentheses

Ten naïve male and 10 naïve female B6 mice were assessed for differences in both CB_1_R and CB_2_R gene expression in the PAG, cerebellum, hippocampus, and spinal cord (Fig. 5). Comparing gene expression across sex, males had significantly greater CB_1_R expression (0.1414 + 0.04594) compared to female (0.02594 + 0.009844) littermates (*t*_18_=2.457, *p*=0.0244) in the PAG while females displayed greater CB_1_R expression in the hippocampus (0.2083 + 0.03462) relative to their male (0.04177 + 0.01121) B6 counterparts (*t*_18_=4.577, *p*=0.0002). There was no difference in CB_1_R expression as a function of sex in either the cerebellum (*p*=0.3884) or spinal cord (*p*=0.9523). Results assessing CB_2_R expression revealed it was not detected in any of the brain regions examined (Table 3).

**Table 3:** Absolute values of calculated CB_1_R and CB_2_R mRNA expression in male and female B6 and CBA mice

Region	Expression	B6 mouse	CBA mouse
PAG	CB_1_R male CB_1_R female	0.1414 (0.04594)* 0.02594 (0.009844)	0.01045 (0.002193) 0.05805 (0.02382)
	CB_2_R male CB_2_R female	Not detected Not detected	Not detected Not detected
Hippocampus	CB_1_R male CB_1_R female	0.04177 (0.01121)*** 0.2083 (0.03462)	0.01309 (0.004749) 0.01476 (0.006017)
	CB_2_R male CB_2_R female	Not detected Not detected	Not detected Not detected
Cerebellum	CB_1_R male CB_1_R female	0.1501 (0.0586) 0.08873 (0.03723)	0.03471 (0.003525) 0.02839 (0.002685)
	CB_2_R male CB_2_R female	Not detected Not detected	Not detected Not detected
Spinal Cord	CB_1_R male CB_1_R female	0.05607 (0.02142) 0.05764 (0.01456)	0.006439 (0.000724) 0.005561 (0.00004016)
	CB_2_R male CB_2_R female	Not detected Not detected	Not detected Not detected

Values shown are mean CB_1_R and CB_2_R gene expression and (standard error) in male and female B6 and CBA mice in the periaqueductal grey (PAG), hippocampus, cerebellum, and spinal cord

*Not Detected* indicated instances where expression was not detectable

*p<0.05; ***p<0.001 in absolute gene expression between males and females within the same mouse line

#### CBA mice

CB_1_R and CB_2_R gene expression in the PAG, cerebellum, hippocampus, and spinal cord was assessed in seven naïve male and female CBA mice (Fig. 5). While there were no sex differences in CB_1_R expression in the hippocampus (*p=*0.8313), cerebellum (*p*=0.1792) or spinal cord (*p*=0.3098), females trended towards showing greater CB_1_R expression in the PAG (0.05805 + 0.02382) compared to male (0.01045 + 0.002193) littermates (*p*=0.0698). When using the ROUT method with the coefficient *Q* set to 1% to identify outliers, there was one female outlier which, upon removal, resulted in female CBA mice having significantly greater CB_1_R expression in the PAG compared to male littermates (*t*_11_=3.243, *p*=0.0078). Like CB_2_R expression in B6 mice, CB_2_R expression was not detectable in any brain region in CBA mice (Table 3).

## Discussion

The primary aim of this paper was to determine whether sex differences in Δ^9^-THC-induced antinociception and tolerance in B6 mice are consistent across multiple mouse strains. We also sought to determine whether sex differences in the antinociceptive responses to Δ^9^-THC in the B6 strain persist with age and/or may be contingent upon differences in CB_1_R and/or CB_2_R mRNA expression in regions associated with acute pain, including PAG and spinal cord. Consistent with our previous findings, we found that B6 males were more sensitive to the antinociceptive effects of Δ^9^-THC than their female counterparts. Interestingly, we found that our sex differences in Δ^9^-THC-induced antinociception are strain specific and that there was considerable variability in Δ^9^-THC response across the strains assessed. However, one consistency across strains was that female mice of all strains developed tolerance to the antinociceptive effects of Δ^9^-THC faster than male littermates. Finally, we determined that CB_1_R mRNA expression in the PAG (but not the spinal cord) is decreased in female B6 mice relative to males and may partially explain the observed B6 sex differences in Δ^9^-THC-induced antinociception.

Our findings that male B6 mice are more sensitive to the antinociceptive (but not hypothermic) effects of Δ^9^-THC are consistent with previous findings in our lab (Henderson-Redmond et al. 2021, 2022; Lafleur et al. 2018). In contrast, most preclinical rat studies have shown female rats to be more sensitive to cannabinoid-induced antinociception (Craft et al. 2012; Romero et al. 2002; Tseng and Craft 2001; Wakley et al. 2014b; Wakley and Craft 2011; Wiley et al. 2021) and the cannabimimetic effects of synthetic cannabinoids (Wiley et al. 2017) than their male counterparts. While most of these studies were performed using Sprague-Dawley rats (Craft et al. 2012; Tseng and Craft 2001; Wakley and Craft 2011; Wakley et al. 2014b; Wiley et al. 2017, 2021), this increased cannabinoid response in females was also observed in Wistar rats (Romero et al. 2002). In contrast, considerably less is known about whether sex differences in Δ^9^-THC-induced antinociception in B6 mice are observed in other mouse strains.

One prior study examining sex- and/or strain-specific effects of Δ^9^-THC found that male B6 and DBA mice were more sensitive to the ataxic effects of 10 mg/kg Δ^9^-THC than female mice and that, overall, B6 mice were more sensitive to the ataxic effects of 10 mg/kg THC than DBA mice (Parks et al. 2020). While no sex differences were observed in acute Δ^9^-THC-induced antinociception in B6 or DBA mice (Parks et al. 2020), there was a significant strain difference with DBA mice showing increased Δ^9^-THC-induced antinociception compared to B6 mice following 10 mg/kg Δ^9^-THC. Interestingly, comparison of the ED_50_ values for Δ^9^-THC-induced antinociception in male and female B6 and DBA mice tested as part of our study determined that while male B6 and DBA mice did not differ from each other (B6 ED_50_=59.37 versus DBA ED_50_=62.01), female DBA mice were much more sensitive to the antinociceptive effects of Δ^9^-THC (ED_50_=36.8) compared to B6 female mice (ED_50_=298.4; Table 1). Likewise, Parks et al. (2020) found that females of both species were faster to develop tolerance to the antinociceptive effects of Δ^9^-THC than their male counterparts, consistent with our results for both B6 and DBA mice in this study.

The strains of mice chosen for this study were based off a prior study examining sex differences in acute morphine antinociception across 11 inbred mouse strains (Kest et al. 1999). Of the 11 strains of mice tested, males were more sensitive to morphine than females for 3 strains (AKR, C57BL/6, and SWR) while female mice from one strain (CBA) were more sensitive than males for morphine-induced antinociception. In contrast, there were no sex differences among the remaining 7 mouse strains, including DBA/J mice (Kest et al. 1999). Morphine exerts most of its antinociceptive effects through mu-opioid receptors (Rossi et al. 1994) which, like CB_1_Rs, are G_i_/G_o_ coupled GPCRs that inhibit adenylyl cyclase activity (Howlett 1985), activate mitogen-activated protein kinases (MAPK; Bouaboula et al. 1995) and inwardly rectifying K^+^ channels (Mackie et al. 1995), and inhibit voltage gated Ca^2+^ channels (Law et al. 2000; Mackie et al. 1993). As such, we hypothesized similar sex differences might exist for Δ^9^-THC-induced antinociception amongst these same lines. And while we did find similar results for B6 and DBA mice, and to some extent CBA mice, we did not find sex differences in Δ^9^-THC-induced antinociception among AKR mice.

Unlike morphine, where AKR mice showed clear dose-dependent increases in morphine-induced antinociception, male and female AKR mice failed to show much Δ^9^-THC-induced antinociception at all (Fig. 1c). It is possible that higher doses of Δ^9^-THC might be required in AKR mice to elicit antinociceptive responses; however, these same doses of Δ^9^-THC elicited pronounced hypothermic responses (Fig. 2c), suggesting that our lack of an antinociceptive response is not due to altered Δ^9^-THC pharmacokinetics in these mice. Other methodological differences between our work and Kest’s that could result in different findings in AKR mice can include differences in route of administration and antinociceptive assay utilized: centrally (ICV) administered morphine versus peripherally (IP) administered Δ^9^-THC and the tail-withdrawal test (supraspinal) versus tail-flick (spinally mediated) assays. The consistency between our findings on Δ^9^-THC-induced antinociception and previous studies investigating sex differences in morphine response indicates that robust strain and sex differences exist for drug-induced antinociception, and these should be carefully considered when assessing drug-induced antinociception.

Sex differences in Δ^9^-THC-induced antinociception could be attributable to differences in sex hormone signaling, or interactions between sex hormones and the endocannabinoid system. For example, estrogen can interfere with ∆^9^-THC’s ability to bind to CB_1_R (Wakley et al. 2014a) while testosterone may have protective effects on inflammation (Jayaraman et al. 2014; Klein and Flanagan 2016; Roglio et al. 2007). Previous work in rats determined that endocannabinoid levels fluctuate across the estrous cycle in several brain regions (Bradshaw et al. 2006; de Fonseca et al. 1994a; González et al. 2000) and that hormone fluctuations across the estrus cycle can alter the efficacy of G protein coupling to CB_1_R (Riebe et al. 2010) without altering CB_1_R density (Farquhar et al. 2019). Although our studies did not track mice across estrus cycle, we did assess differences in Δ^9^-THC-induced antinociception in younger (3 month) and older (18 month) B6 mice to determine if the sex differences observed in younger, cycling mice persist in older, non-cycling female mice. According to the Jackson Laboratory, mice that are 3 months of age correspond to humans that are ~20-30 years while 18-month-old mice corresponds to humans in old age (56-69 years old). Notably, the sex differences observed in younger B6 mice were absent in older B6 mice. However, older B6 mice were more sensitive across the same range of Δ^9^-THC doses than their younger counterparts, suggesting that age and sex hormones may affect ∆^9^-THC-induced antinociception and tolerance in mice (for a review see Piscura et al. 2023a).

Differences in CB_1_R density, downregulation, and/or desensitization are all mechanisms that have been proposed as potential reasons for observed sex differences in cannabinoid sensitivity and/or tolerance. Previous work has also found differences in CB_1_R density and desensitization between male and female rodents (Castelli et al. 2014; de Fonseca et al. 1994b; Farquhar et al. 2019; González et al. 2005 but see Wiley et al. 2021). It is possible that sex differences in cannabinoid response are also response specific. For example, the observation of sex differences in Δ^9^-THC-induced antinociception, these sex differences did not extend to the hypothermic response to cannabinoids. This raises the possibility that response- and sex-specific differences in acute cannabinoid response might be due to underlying differences in cannabinoid signaling within the specific brain regions such as the PAG, spinal cord, and hypothalamus that mediate these responses. Although there are no described sex differences in CB_1_R densities and coupling in many regions of the mouse brain, including the cerebellum (Farquhar et al. 2019; Wiley et al. 2021), very little is known about possible sex differences in CB_1_R (or CB_2_R) levels in areas that control antinociception.

There is evidence suggesting that sex differences in Δ^9^-THC-induced antinociception may be attributed to differences in the relative expression of CB_1_R and CB_2_R between male and female rats (Craft et al. 2012). Previously, we determined that Δ^9^-THC-induced hypothermia in both sexes and that Δ^9^-THC-induced antinociception in males was exclusively mediated via CB_1_Rs. However, the role of CB_1_R versus CB_2_R in mediating Δ^9^-THC-induced antinociception in females was less clear, in part due to the modest antinociceptive response to Δ^9^-THC in females (Henderson-Redmond et al. 2022). Since Δ^9^-THC acts as a mixed CB_1_R/CB_2_R agonist, we examined whether differences in CB_1_R and CB_2_R gene expression may explain observed sex differences in antinociception. Because B6 and CBA mice showed evidence of opposing sex differences in Δ^9^-THC-induced antinociception, we examined naïve male and female B6 and CBA mice for differences in CB_1_R and CB_2_R gene expression in brain regions responsible for Δ^9^-THC-induced antinociception, such as the PAG and spinal cord. We found that in naïve mice, B6 males exhibited increased CB_1_R gene expression compared to females in the PAG while the opposite was found in CBA mice. These differences paralleled the sex differences in Δ^9^-THC-induced antinociception observed in this study. Interestingly, there were no differences in CB_1_R gene expression found in the spinal cord, suggesting that the PAG may mediate sex differences in Δ^9^-THC-induced antinociception. While differences in CB_1_R gene expression do not directly translate into increased CB_1_R surface expression, the presence of increased CB_1_R gene expression and mRNA means there is the potential for more CB_1_Rs to be translated. In contrast, no CB_2_R gene expression was detected (Table 3), suggesting that the strain dependent sex differences are likely due to differences in CB_1_R.

While Kest’s study did not assess tolerance to morphine, following 7 days of once-daily treatment with 30 mg/kg of Δ^9^-THC, we found that mice from all strains developed tolerance to the antinociceptive and hypothermic effects of Δ^9^-THC. In B6 and DBA mice, female mice acquired greater tolerance to the antinociceptive effects of Δ^9^-THC than males (Table 1). This finding of faster cannabinoid tolerance in females is consistent with other studies in both rats (Nguyen et al. 2018, 2020; Wakley et al. 2014b) and mice (Henderson-Redmond et al. 2021, 2022; Parks et al. 2020) and occurred using paradigms examining the same dose administration and the use of equally efficacious doses (Henderson-Redmond et al. 2021; Wakley et al. 2014b). Interestingly, female (but not male) Wistar rats developed tolerance to Δ^9^-THC following vapor inhalation (Nguyen et al. 2018, 2020), suggesting that this effect could be even more pronounced in a more clinically relevant model of Δ^9^-THC administration. Future studies examining sex differences and cannabis-related tolerance in mice should utilize vapor or oral administration models as they are more clinically relevant than the model of IP administration used in the current study. Likewise, in addition to assessing estrus cycle, bloods should also be taken to be able to assess differences in Δ^9^-THC and metabolite levels.

While clinical studies examining sex differences in cannabis tolerance is limited, evidence seems to suggest that women develop tolerance to cannabis faster than men. For example, women who were regular users of cannabis were less sensitive to the antinociceptive effects of cannabis than men in the cold pressor test (Cooper and Haney 2016). Similarly, men and women who were not cannabis users both showed evidence of tachycardia following first usage of Δ^9^-THC. Following the second usage, tachycardia was less pronounced in women than men, suggesting that tolerance to the cardiovascular effects of Δ^9^-THC occurs more rapidly in women (Cocchetto et al. 1981). Further, despite men having an increased lifetime use and a greater incidence of developing CUDs, women display an accelerated advancement (also called telescoping) from first usage to CUD diagnosis (Farmer et al. 2015; Kerridge et al. 2018). This “telescoping” effect suggests that women display more rapid tolerance development to cannabis than men (for a review, see Towers et al. 2022). Specifically, women seem to progress faster to seek treatment for CUDs than men (Ehlers et al. 2010; Hernandez-Avila et al. 2004; Lewis et al. 2014) and show an escalated usage from initial use of cannabis to dependence (Ehlers et al. 2010; Kerridge et al. 2018; Khan et al. 2013) compared to men. Thus, regardless of strain, female mice showing a propensity to faster tolerance development to Δ^9^-THC-mediated antinociception are seemingly congruent with the limited clinical studies examining sex differences in cannabis tolerance, suggesting that sex should be an important consideration.

In this study, male and female mice of four different inbred mouse strains were assessed for sex differences in Δ^9^-THC-induced antinociception and hypothermia and subsequent tolerance development. While females of all strains were faster to develop tolerance to the antinociceptive effects of Δ^9^-THC, only two strains, B6 and CBA mice, showed any sex differences in Δ^9^-THC-induced antinociception with B6 males and CBA females showing increased responsiveness to Δ^9^-THC than their respective littermates. Subsequent examination of CB_1_R and CB_2_R mRNA expression in naïve B6 and CBA mice revealed that sex differences in CB_1_R mRNA expression in the PAG may potentially explain the observed differences in acute Δ^9^-THC-induced antinociception in these mice. Taken together, these data suggest that not only is it important to consider what strain to use when assessing sex differences in cannabinoids response in mice but that similarly, when assessing the potential efficacy of cannabinoid-based therapies in clinical populations, differences in sex and genetics should be considered.

## Supplementary information

ESM 1 (PDF 507 kb)

## Data Availability

All data will be made available upon request.

## Abbreviations

ANOVA: Analyses of variance
βArr2: Beta-arrestin 2
CB: Cannabinoid
CB_1_/CB_1_R: Type-1 cannabinoid receptor
CB_2_/CB_2_R: Type-2 cannabinoid receptor
CI: Confidence interval
CUD: Cannabinoid use disorder(s)
∆^9^-THC: Delta-9-tetrahydrocannabinol
ED_50_: Mean effective dose
GPCR: G protein-coupled receptor
GRK: G protein-coupled receptor kinase
ICV: Intracerebroventricular
IP: Intraperitoneal
mRNA: Messenger ribonucleic acid
MAPK: Mitogen-activated protein kinase
MOR: Mu opioid receptor
%∆BT: Percent change in body temperature
%MPE: Percentage of maximal possible effect
PAG: Periaqueductal grey
SC: Spinal cord
SEM: Standard error of the mean

## References

[CR1] Anderson BM, Rizzo M, Block RI (2010). Sex differences in the effects of marijuana on simulated driving performance. J Psychoactive Drugs.

[CR2] Bouaboula M, Bourrié B, Rinaldi-Carmona M (1995). Stimulation of cannabinoid receptor CB1 induces krox-24 expression in human astrocytoma cells. J Biol Chem.

[CR3] Boudreau D, Von Korff M, Rutter CM (2009). Trends in long-term opioid therapy for chronic non-cancer pain. Pharmacoepidemiol Drug Saf.

[CR4] Bradshaw HB, Rimmerman N, Krey JF, Walker JM (2006). Sex and hormonal cycle differences in rat brain levels of pain-related cannabimimetic lipid mediators. Am J Physiol Regul Integr Comp Physiol.

[CR5] Campbell CI, Weisner C, LeResche L (2010). Age and gender trends in long-term opioid analgesic use for noncancer pain. Am J Public Health.

[CR6] Castelli M, Fadda P, Casu A (2014). Male and female rats differ in brain cannabinoid CB1 receptor density and function and in behavioural traits predisposing to drug addiction: effect of ovarian hormones. Curr Pharm Des.

[CR7] Cocchetto DM, Owens SM, Perez-Reyes M (1981). Relationship between plasma delta-9-tetrahydrocannabinol concentration and pharmacologic effects in man. Psychopharmacology (Berl).

[CR8] Cooper ZD, Haney M (2014). Investigation of sex-dependent effects of cannabis in daily cannabis smokers. Drug Alcohol Depend.

[CR9] Cooper ZD, Haney M (2016). Sex-dependent effects of cannabis-induced analgesia. Drug Alcohol Depend.

[CR10] Copersino ML, Boyd SJ, Tashkin DP (2006). Quitting among non-treatment-seeking marijuana users: reasons and changes in other substance use. Am J Addict.

[CR11] Craft RM, Wakley AA, Tsutsui KT, Laggart JD (2012). Sex differences in cannabinoid 1 vs. cannabinoid 2 receptor-selective antagonism of antinociception produced by 9-tetrahydrocannabinol and CP55,940 in the rat. J Pharmacol Exp Ther.

[CR12] Cuttler C, Mischley LK, Sexton M (2016). Sex differences in cannabis use and effects: a cross-sectional survey of cannabis users. Cannabis Cannabinoid Res.

[CR13] Dahlhamer J, Lucas J, Zelaya C (2018). Prevalence of chronic pain and high-impact chronic pain among adults — United States, 2016. MMWR Morb Mortal Wkly Rep.

[CR14] de Fonseca FR, Cebeira M, Ramos JA (1994). Cannabinoid receptors in rat brain areas: sexual differences, fluctuations during estrous cycle and changes after gonadectomy and sex steroid replacement. Life Sci.

[CR15] de Fonseca FR, Gorriti MA, Fernández-Ruiz JJ (1994). Downregulation of rat brain cannabinoid binding sites after chronic Δ9-tetrahydrocannabinol treatment. Pharmacol Biochem Behav.

[CR16] Ehlers CL, Gizer IR, Vieten C (2010). Cannabis dependence in the San Francisco Family Study: age of onset of use, DSM-IV symptoms, withdrawal, and heritability. Addict Behav.

[CR17] Farmer RF, Kosty DB, Seeley JR (2015). Natural course of cannabis use disorders. Psychol Med.

[CR18] Farquhar CE, Breivogel CS, Gamage TF (2019). Sex, THC, and hormones: effects on density and sensitivity of CB 1 cannabinoid receptors in rats. Drug Alcohol Depend.

[CR19] González S, Bisogno T, Wenger T (2000). Sex steroid influence on cannabinoid CB1 receptor mRNA and endocannabinoid levels in the anterior pituitary gland. Biochem Biophys Res Commun.

[CR20] González S, Cebeira M, Fernández-Ruiz J (2005). Cannabinoid tolerance and dependence: a review of studies in laboratory animals. Pharmacology Biochemistry and Behavior.

[CR21] Haney M (2007). Opioid Antagonism of cannabinoid effects: differences between marijuana smokers and nonmarijuana smokers. Neuropsychopharmacology.

[CR22] Henderson-Redmond AN, Crawford LTC, Sepulveda DE (2021). Sex differences in tolerance to delta-9-tetrahydrocannabinol in mice with cisplatin-evoked chronic neuropathic pain. Front Mol Biosci.

[CR23] Henderson-Redmond AN, Nealon CM, Davis BJ (2020). c-Jun N terminal kinase signaling pathways mediate cannabinoid tolerance in an agonist-specific manner. Neuropharmacology.

[CR24] Henderson-Redmond AN, Sepulveda DE, Ferguson EL (2022). Sex-specific mechanisms of tolerance for the cannabinoid agonists CP55,940 and delta-9-tetrahydrocannabinol (Δ9-THC). Psychopharmacology (Berl).

[CR25] Hernandez-Avila CA, Rounsaville BJ, Kranzler HR (2004). Opioid-, cannabis- and alcohol-dependent women show more rapid progression to substance abuse treatment. Drug Alcohol Depend.

[CR26] Howlett AC (1985). Cannabinoid inhibition of adenylate cyclase. Biochemistry of the response in neuroblastoma cell membranes. Mol Pharmacol.

[CR27] Jayaraman A, Lent-Schochet D, Pike CJ (2014). Diet-induced obesity and low testosterone increase neuroinflammation and impair neural function. J Neuroinflammation.

[CR28] Kerridge BT, Pickering R, Chou P (2018). DSM-5 cannabis use disorder in the National Epidemiologic Survey on Alcohol and Related Conditions-III: gender-specific profiles. Addict Behav.

[CR29] Kest B, Wilson SG, Mogil JS (1999). Sex differences in supraspinal morphine analgesia are dependent on genotype. J Pharmacol Exp Ther.

[CR30] Khan SS, Secades-Villa R, Okuda M (2013). Gender differences in cannabis use disorders: results from the National Epidemiologic Survey of Alcohol and Related Conditions. Drug Alcohol Depend.

[CR31] Klein SL, Flanagan KL (2016). Sex differences in immune responses. Nat Rev Immunol.

[CR32] Kolodny A, Courtwright DT, Hwang CS (2015). The prescription opioid and heroin crisis: a public health approach to an epidemic of addiction. Annu Rev Public Health.

[CR33] Lafleur RA, Wilson RP, Morgan DJ, Henderson-Redmond AN (2018). Sex differences in antinociceptive response to Δ-9-tetrahydrocannabinol and CP 55,940 in the mouse formalin test. Neuroreport.

[CR34] Law PY, Wong YH, Loh HH (2000). Molecular mechanisms and regulation of opioid receptor signaling. Annu Rev Pharmacol Toxicol.

[CR35] Lewis B, Hoffman LA, Nixon SJ (2014). Sex differences in drug use among polysubstance users. Drug Alcohol Depend.

[CR36] Livak KJ, Schmittgen TD (2001). Analysis of relative gene expression data using real-time quantitative PCR and the 2-ΔΔCT method. Methods.

[CR37] Mackie K, Devane WA, Hille B (1993). Anandamide, an endogenous cannabinoid, inhibits calcium currents as a partial agonist in N18 neuroblastoma cells. Mol Pharmacol.

[CR38] Mackie K, Lai Y, Westenbroek R, Mitchell R (1995). Cannabinoids activate an inwardly rectifying potassium conductance and inhibit Q-type calcium currents in AtT20 cells transfected with rat brain cannabinoid receptor. J Neurosci.

[CR39] Matsuda LA, Lolait SJ, Brownstein MJ (1990). Structure of a cannabinoid receptor and functional expression of the cloned cDNA. Nature.

[CR40] Moore CF, Weerts EM (2021). Cannabinoid tetrad effects of oral Δ9-tetrahydrocannabinol (THC) and cannabidiol (CBD) in male and female rats: sex, dose-effects and time course evaluations. Psychopharmacology (Berl).

[CR41] Morgan DJ, Davis BJ, Kearn CS (2014). Mutation of putative GRK phosphorylation sites in the cannabinoid receptor 1 (CB1R) confers resistance to cannabinoid tolerance and hypersensitivity to cannabinoids in mice. J Neurosci.

[CR42] Mücke M, Phillips T, Radbruch L (2018). Cannabis-based medicines for chronic neuropathic pain in adults. Cochrane Database Syst Rev.

[CR43] Munro S, Thomas KL, Abu-Shaar M (1993). Molecular characterization of a peripheral receptor for cannabinoids. Nature.

[CR44] Nahin RL (2015). Estimates of Pain Prevalence and Severity in Adults: United States, 2012. J Pain.

[CR45] National Research Council (2011) Guide for the care and use of laboratory animals - Committee for the Update of the Guide for the Care and Use of Laboratory Animals, Institute for Laboratory Animal Research. Guide for the care and use of laboratory animals 327:220

[CR46] Nguyen JD, Creehan KM, Kerr TM, Taffe MA (2020). Lasting effects of repeated ∆9-tetrahydrocannabinol vapour inhalation during adolescence in male and female rats. Br J Pharmacol.

[CR47] Nguyen JD, Grant Y, Kerr TM (2018). Tolerance to hypothermic and antinoceptive effects of ∆9-tetrahydrocannabinol (THC) vapor inhalation in rats. Pharmacol Biochem Behav.

[CR48] Nguyen PT, Schmid CL, Raehal KM (2012). β-Arrestin2 regulates cannabinoid CB1 receptor signaling and adaptation in a central nervous system region–dependent manner. Biol Psychiatry.

[CR49] Parks C, Jones BC, Moore BM, Mulligan MK (2020). Sex and strain variation in initial sensitivity and rapid tolerance to Δ9-tetrahydrocannabinol. Cannabis Cannabinoid Res.

[CR50] Penetar DM, Kouri EM, Gross MM (2005). Transdermal nicotine alters some of marihuana’s effects in male and female volunteers. Drug Alcohol Depend.

[CR51] Piscura MK, Henderson-Redmond AN, Barnes RC (2023). Mechanisms of cannabinoid tolerance. Biochem Pharmacol.

[CR52] Piscura MK, Sepulveda DE, Maulik M (2023). Cannabinoid tolerance in S426A/S430A x β-arrestin 2 knockout double-mutant mice. J Pharmacol Exp Ther.

[CR53] Riebe CJN, Hill MN, Lee TTY (2010). Estrogenic regulation of limbic cannabinoid receptor binding. Psychoneuroendocrinology.

[CR54] Roglio I, Bianchi R, Giatti S (2007). Testosterone derivatives are neuroprotective agents in experimental diabetic neuropathy. Cell Mol Life Sci.

[CR55] Romero EM, Fernández B, Sagredo O (2002). Antinociceptive, behavioural and neuroendocrine effects of CP 55,940 in young rats. Dev Brain Res.

[CR56] Roser P, Gallinat J, Weinberg G (2009). Psychomotor performance in relation to acute oral administration of Δ9-tetrahydrocannabinol and standardized cannabis extract in healthy human subjects. Eur Arch Psychiatry Clin Neurosci.

[CR57] Rossi G, Pan YX, Cheng J, Pasternak GW (1994). Blockade of morphine analgesia by an antisense oligodeoxynucleotide against the mu receptor. Life Sci.

[CR58] Sim LJ, Hampson RE, Deadwyler SA, Childers SR (1996). Effects of chronic treatment with 9-tetrahydrocannabinol on cannabinoid-stimulated [35S]GTPS autoradiography in rat brain. J Neurosci.

[CR59] Towers EB, Williams IL, Qillawala EI (2022). Sex/gender differences in the time-course for the development of substance use disorder: a focus on the telescoping effect. Pharmacol Rev.

[CR60] Tseng AH, Craft RM (2001). Sex differences in antinociceptive and motoric effects of cannabinoids. Eur J Pharmacol.

[CR61] Vowles KE, McEntee ML, Julnes PS (2015). Rates of opioid misuse, abuse, and addiction in chronic pain. Pain.

[CR62] Wakley AA, Craft RM (2011). Antinociception and sedation following intracerebroventricular administration of Δ9-tetrahydrocannabinol in female vs. male rats. Behav Brain Res.

[CR63] Wakley AA, McBride AA, Vaughn LK, Craft RM (2014). Cyclic ovarian hormone modulation of supraspinal Δ9- tetrahydrocannabinol-induced antinociception and cannabinoid receptor binding in the female rat. Pharmacol Biochem Behav.

[CR64] Wakley AA, Wiley JL, Craft RM (2014). Sex differences in antinociceptive tolerance to delta-9-tetrahydrocannabinol in the rat. Drug Alcohol Depend.

[CR65] Wardle MC, Marcus BA, De Wit H (2015). A preliminary investigation of individual differences in subjective responses to D-amphetamine, alcohol, and delta-9-tetrahydrocannabinol using a within-subjects randomized trial. PLoS One.

[CR66] Wetherill RR, Jagannathan K, Hager N (2015). Sex differences in associations between cannabis craving and neural responses to cannabis cues: implications for treatment. Exp Clin Psychopharmacol.

[CR67] Wiley JL, Barrus DG, Farquhar CE (2021). Sex, species and age: effects of rodent demographics on the pharmacology of ∆9-tetrahydrocanabinol. Prog Neuropsychopharmacol Biol Psychiatry.

[CR68] Wiley JL, Lefever TW, Marusich JA, Craft RM (2017). Comparison of the discriminative stimulus and response rate effects of Δ9-tetrahydrocannabinol and synthetic cannabinoids in female and male rats. Drug Alcohol Depend.

[CR69] Yong RJ, Mullins PM, Bhattacharyya N (2022). Prevalence of chronic pain among adults in the United States. Pain.

