# Supplementary material for: Sex differences in acute delta-9-tetrahydrocannabinol (Δ9-THC) response and tolerance as a function of mouse strain
Source: Psychopharmacology (Berl). 2023 Jul 29;240(9):1987–2003. doi: 10.1007/s00213-023-06421-8 (PMC10471687; doi:10.1007/s00213-023-06421-8)
Supplement: Supplementary file 1 — (PDF 507 kb) [file 213_2023_6421_MOESM1_ESM.pdf]

Manuscript Title: Sex differences in acute delta-9-tetrahydrocannabinol (Δ^9-THC) response and tolerance as a function of mouse strain

Authors: Courtney F Lulek, Malabika Maulik, Swarup Mitra, Josée Guindon, Daniel J Morgan, Angela N Henderson-Redmond

Journal: Psychopharmacology

Affiliation Corresponding Author: Department of Biomedical Sciences, Marshall University, Huntington, WV 25755

Corresponding Author email: redmonda@marshall.edu

Supplementary Table 1 Sex-differences in acute antinociception and hypothermia in B6 mice receiving vehicle

Data represent the Means and SEM (standard error of the mean) in Percent Maximum Possible Effect (%MPE) for Tail Flick (A) and Percent Change in Body Temperature (%ΔBT) for Hypothermia (B) in B6 Mice. For the first two columns (PRE VEH), the mean and SEM for each sex is given for each dose following the administration of vehicle (VEH only) with the p value comparing sex within each dose listed to the right. For the next set of columns, the mean and SEM for mice getting THC (0-100 mg/kg; PRE THC) is listed with the P value to the right comparing sex within each dose with subsequent sets of p values comparing naïve mice getting VEH to those getting THC within each sex. Following seven days of once-daily treatment with vehicle (VEH) or 30 mg/kg THC, all mice were tested across a range of doses (0-130 mg/kg) of THC to generate post (POST) dose-response curves. The mean and SEM for each dose is listed for mice that received vehicle for 7 days prior to being assessed for the post dose-response curve of THC. P values assess sex differences in the post dose-response curve within each dose and within each sex compare differences between Pre and Post curves in VEH mice. *Significant differences are italicized; lines denote instances where values or comparisons are not applicable

1A. B6 Tail-flick

Dose	**PRE VEH Males (N=5)**	**PRE VEH Females (N=5)**	**PRE VEH/Sex P value**	**Pre THC Males (N=12)**	**Pre THC Females (N=13)**	**PRE THC/Sex P value**	**PRE Male VEH/THC P value**	**PRE Female VEH/THC P value**	**Post VEH Males (N=2)**	**Post VEH Females (N=2)**	**POST VEH/Sex P value**	**Pre/Post Male VEH P value**	**Pre/Post Female VEH P value**
0	-3.828 (2.629)	-2.182 (2.622)	>0.9999	-0.903 (2.488)	1.031 (1.483)	>0.9999	>0.9999	>0.9999	-7.590 (10.790)	-1.860 (2.500)	>0.9999	>0.9999	>0.9999
1	-4.924 (4.185)	-1.748 (2.511)	>0.9999	3.544 (3.019)	0.618 (2.259)	>0.9999	>0.9999	>0.9999	—	—	—	—	—
3	-1.120 (1.979)	1.740 (1.175)	>0.9999	11.177 (4.953)	5.085 (2.245)	>0.9999	>0.9999	>0.9999	18.830 (2.520)	9.270 (2.250)	>0.9999	0.6163	0.9608
10	-2.676 (3.261)	3.288 (3.909)	>0.9999	29.928 (6.902)	16.006 (2.640)	0.2655	*0.0060**	0.2757	46.545 (31.455)	19.610 (2.370)	>0.9999	*0.0029**	*0.0378**
30	-2.674 (3.597)	1.314 (2.719)	>0.9999	41.128 (7.536)	22.395 (3.182)	*0.0428**	*<0.0001**	*0.0069**	67.060 (32.940)	32.480 (4.050)	0.7002	*<0.0001**	*<0.0001**
100	0.370 (5.021)	2.088 (1.941)	>0.9999	55.366 (8.052)	33.946 (7.294)	*0.0131**	*<0.0001**	*<0.0001**	100.00 (0.000)	74.085 (13.535)	>0.9999	*<0.0001**	*<0.0001**
130	—	—	—	—	—	—	—	—	100.00 (0.000)	90.175 (9.825)	>0.9999	—	—
Mean	-2.475 (0.7711)	0.75 (0.9012)	0.4134	23.37 (9.152)	13.18 (5.462)	*0.0345**	*0.0017**	*0.0042**	54.14 (17.78)	37.29 (15.06)	0.2137	*<0.0001**	*0.0107**
Sum	-14.85	4.50		140.2	79.08				324.8	223.8			

1B. B6 Hypothermia

Dose	**PRE VEH Males (N=5)**	**PRE VEH Females (N=5)**	**PRE VEH/Sex P value**	**Pre THC Males (N=12)**	**Pre THC Females (N=13)**	**PRE THC/Sex P value**	**PRE Male VEH/THC P value**	**PRE Female VEH/THC P value**	**Post VEH Males (N=2)**	**Post VEH Females (N=2)**	**POST VEH/Sex P value**	**Pre/Post Male VEH P value**	**Pre/Post Female VEH P value**
0	-1.752 (0.554)	0.484 (0.491)	0.1697	-1.526 (0.308)	-0.016 (0.334)	>0.9999	>0.9999	>0.9999	1.355 (1.615)	0.270 (0.270)	>0.9999	0.1727	>0.9999
1	-1.856 (0.629)	-0.310 (0.648)	0.7466	-0.783 (0.335)	-0.136 (0.456)	>0.9999	>0.9999	>0.9999	___	___	___	___	___
3	-2.758 (0.556)	-0.840 (0.984)	0.3495	-2.021 (0.568)	-1.273 (0.486)	>0.9999	>0.9999	>0.9999	-3.080 (1.510)	1.485 (0.675)	0.8511	>0.9999	0.9266
10	-3.444 (0.769)	-1.108 (0.736)	0.1334	-8.246 (1.229)	-7.288 (1.050)	>0.9999	*0.0133**	*0.0002**	-14.145 (2.075)	-10.695 (2.315)	>0.9999	*<0.0001**	*<0.0001**
30	-2.968 (0.592)	-2.394 (0.706)	>0.9999	-12.345 (1.265)	-11.810 (1.181)	>0.9999	*<0.0001**	*<0.0001**	-13.845 (0.065)	-11.630 (2.690)	>0.9999	*<0.0001**	*<0.0001**
100	-3.066 (0.977)	-1.810 (0.536)	>0.9999	-15.475 (1.228)	-15.863 (1.042)	>0.9999	*<0.0001**	*<0.0001**	-17.440 (0.410)	-17.190 (3.680)	>0.9999	*<0.0001**	*<0.0001**
130	___	___	___	___	___	___	___	____	-16.645 (0.155)	-18.950 (3.810)	>0.9999	___	___
Mean	-2.641 (0.28)	-0.9963 (0.4209)	0.0813	-6.733 (2.549)	-6.064 (2.74)	0.4456	*0.0021**	*<0.0001**	-10.63 (3.193)	-9.452 (3.514)	0.4513	*0.0011**	*0.0021**
Sum	-15.84	-5.978		-40.4	-36.39				-63.8	-56.71			

Supplementary Table 2 Sex-differences in acute antinociception and hypothermia in DBA mice receiving vehicle

Data represent the Means and SEM (standard error of the mean) in Percent Maximum Possible Effect (%MPE) for Tail Flick (A) and Percent Change in Body Temperature (%ΔBT) for Hypothermia (B) in DBA Mice. For the first two columns (PRE VEH), the mean and SEM for each sex is given for each dose following the administration of vehicle (VEH only) with the p value comparing sex within each dose listed to the right. For the next set of columns, the mean and SEM for mice getting THC (0-100 mg/kg; PRE THC) is listed with the P value to the right comparing sex within each dose with subsequent sets of p values comparing naïve mice getting VEH to those getting THC within each sex. Following seven days of once-daily treatment with vehicle (VEH) or 30 mg/kg THC, all mice were tested across a range of doses (0-130 mg/kg) of THC to generate post (POST) dose-response curves. The mean and SEM for each dose is listed for mice that received vehicle for 7 days prior to being assessed for the post dose-response curve of THC. P values assess sex differences in the post dose-response curve within each dose and within each sex compare differences between Pre and Post curves in VEH mice. *Significant differences are italicized; lines denote instances where values or comparisons are not applicable

2A. DBA Tail-flick

Dose	**PRE VEH Males (N=5)**	**PRE VEH Females (N=5)**	**PRE VEH/Sex P value**	**Pre THC Males (N=10)**	**Pre THC Females (N=10)**	**PRE THC/Sex P value**	**PRE Male VEH/THC P value**	**PRE Female VEH/THC P value**	**Post VEH Males (N=2)**	**Post VEH Females (N=2)**	**POST VEH/Sex P value**	**Pre/Post Male VEH P value**	**Pre/Post Female VEH P value**
0	-3.616 (3.014)	0.104 (1.909)	>0.9999	-2.347 (1.762)	-4.590 (3.029)	0.9997	>0.9999	0.9893	-1.200 (0.360)	-0.840 (1.460)	>0.9999	>0.9999	>0.9999
1	-5.372 (2.210)	-4.448 (1.508)	>0.9999	-6.264 (2.162)	-8.028 (3.029)	>0.9999	>0.9999	0.9975	___	___	___	___	___
3	-1.602 (1.767)	3.732 (1.831)	>0.9999	-1.798 (2.887)	-3.463 (2.805)	>0.9999	>0.9999	>0.9999	46.285 (35.495)	19.150 (9.490)	0.9022	*0.0185**	*0.0187**
10	4.260 (4.910)	6.394 (3.488)	>0.9999	11.403 (2.273)	21.730 (5.931)	0.5588	0.8852	0.2316	65.820 (34.180)	55.170 (2.710)	0.9991	*0.0019**	*<0.0001**
30	9.662 (4.463)	3.620 (3.480)	>0.9999	31.255 (5.409)	49.795 (5.398)	*0.0404**	*0.0144**	*<0.0001**	72.655 (27.345)	67.195 (19.215)	>0.9999	*0.0015**	*<0.0001**
100	4.856 (4.408)	2.938 (2.004)	>0.9999	62.662 (8.283)	69.431 (7.849)	0.8979	*<0.0001**	*<0.0001**	91.610 (8.390)	90.295 (9.705)	>0.9999	*<0.0001**	*<0.0001**
130	___	___	___	___	___	___	___	____	88.480 (11.520)	86.390 (13.610)	>0.9999	___	___
Mean	1.365 (2.369)	0.8127 (1.755)	0.8436	15.82 (10.92)	20.81 (13.26)	0.2086	*0.0069**	*0.0003**	60.61 (14.07)	52.89 (15.01)	0.7559	*0.0075**	*0.0003**
Sum	8.188	4.876		94.91	124.9				363.7	317.4			

2B. DBA Hypothermia

Dose	**PRE VEH Males (N=5)**	**PRE VEH Females (N=5)**	**PRE VEH/Sex P value**	**Pre THC Males (N=10)**	**Pre THC Females (N=10)**	**PRE THC/Sex P value**	**PRE Male VEH/THC P value**	**PRE Female VEH/THC P value**	**Post VEH Males (N=2)**	**Post VEH Females (N=2)**	**POST VEH/Sex P value**	**Pre/Post Male VEH P value**	**Pre/Post Female VEH P value**
0	0.184 (0.813)	0.386 (0.673)	>0.9999	0.083 (0.0416)	-0.045 (0.437)	>0.9999	>0.9999	0.9999	-0.240 (2.130)	1.690 (2.220)	0.9964	0.9998	0.9273
1	-0.194 (0.674)	0.964 (0.770)	0.7228	1.076 (0.422)	1.197 (0.474)	>0.9999	0.9611	>0.9999	___	___	___	___	___
3	-1.532 (0.397)	0.852 (0.318)	0.0530	1.341 (0.389)	1.574 (0.598)	>0.9999	0.3500	0.9979	-3.915 (3.655)	1.955 (1.955)	0.5818	0.6444	0.9631
10	-1.756 (0.213)	0.376 (0.721)	0.1073	-4.020 (1.452)	-4.686 (1.561)	0.9982	0.6246	*0.0092**	-8.700 (2.380)	-1.410 (1.940)	0.3530	*0.0028**	0.7780
30	-0.642 (0.531)	-0.582 (0.307)	>0.9999	-10.505 (1.522)	-13.057 (1.234)	0.4102	*<0.0001**	*<0.0001**	-9.065 (0.145)	-9.865 (2.605)	>0.9999	*0.0003**	*<0.0001**
100	0.108 (1.073)	-1.272 (0.305)	0.5413	-12.127 (1.220)	-13.084 (1.311)	0.9870	*<0.0001**	*<0.0001**	-18.705 (2.915)	-12.330 (3.310)	0.4947	*<0.0001**	*<0.0001**
130	___	___	___	___	___	___	___	____	-18.990 (4.250)	-12.395 (0.455)	0.4584	___	___
Mean	-0.6387 (0.340)	-0.1207 (0.357)	0.1531	-4.025 (2.445)	-4.684 (2.804)	0.4690	*0.0029**	*0.0002**	-9.936 (3.117)	-5.393 (2.812)	0.1310	*0.0003**	*0.0016**
Sum	-3.832	0.724		-24.15	-28.10				-59.62	-32.36			

Supplementary Table 3 Sex-differences in acute antinociception and hypothermia in AKR mice receiving vehicle

Data represent the Means and SEM (standard error of the mean) in Percent Maximum Possible Effect (%MPE) for Tail Flick (A) and Percent Change in Body Temperature (%ΔBT) for Hypothermia (B) in AKR Mice. For the first two columns (PRE VEH), the mean and SEM for each sex is given for each dose following the administration of vehicle (VEH only) with the p value comparing sex within each dose listed to the right. For the next set of columns, the mean and SEM for mice getting THC (0-100 mg/kg; PRE THC) is listed with the P value to the right comparing sex within each dose with subsequent sets of p values comparing naïve mice getting VEH to those getting THC within each sex. Following seven days of once-daily treatment with vehicle (VEH) or 30 mg/kg THC, all mice were tested across a range of doses (0-130 mg/kg) of THC to generate post (POST) dose-response curves. The mean and SEM for each dose is listed for mice that received vehicle for 7 days prior to being assessed for the post dose-response curve of THC. P values assess sex differences in the post dose-response curve within each dose and within each sex compare differences between Pre and Post curves in VEH mice. *Significant differences are italicized; lines denote instances where values or comparisons are not applicable

3A. AKR Tail-flick

Dose	**PRE VEH Males (N=5)**	**PRE VEH Females (N=5)**	**PRE VEH/Sex P value**	**Pre THC Males (N=10)**	**Pre THC Females (N=10)**	**PRE THC/Sex P value**	**PRE Male VEH/THC P value**	**PRE Female VEH/THC P value**	**Post VEH Males (N=4)**	**Post VEH Females (N=4)**	**POST VEH/Sex P value**	**Pre/Post Male VEH P value**	**Pre/Post Female VEH P value**
0	5.256 (4.236)	3.992 (3.456)	0.9998	-2.434 (2.362)	1.180 (1.297)	0.9946	0.8906	0.9983	1.688 (5.315)	-7.840 (11.075)	0.9828	0.9901	0.7606
1	7.648 (2.603)	3.290 (1.965)	0.9366	6.773 (3.189)	0.041 (2.597)	0.8874	>0.9999	0.9961	___	___	___	___	___
3	9.582 (3.948)	2.714 (2.479)	0.6461	11.477 (3.810)	1.793 (2.345)	0.5981	>0.9999	>0.9999	6.173 (5.967)	-2.520 (7.884)	0.9893	0.9943	0.9906
10	4.550 (5.088)	5.394 (1.939)	>0.9999	18.082 (2.611)	14.393 (4.217)	0.9940	0.4143	0.6310	-2.658 (7.212)	-0.950 (13.115)	>0.9999	0.8661	0.9779
30	7.696 (4.889)	4.542 (1.746)	0.9869	13.583 (1.978)	27.535 (4.819)	0.1918	0.9725	*0.0025**	12.938 (6.016)	29.150 (14.002)	0.8150	0.9614	0.0956
100	10.404 (3.156)	7.144 (3.068)	0.9844	39.609 (10.531)	22.975 (6.998)	0.0713	*0.0019**	0.0763	22.898 (6.252)	9.143 (7.021)	0.9036	0.3977	>0.9999
130	___	___	___	___	___	___	___	____	11.015 (8.646)	18.713 (16.179)	0.9944	___	___
Mean	7.568 (0.9201)	4.513 (0.6507)	0.3907	14.52 (5.773)	11.32 (4.930)	0.3103	0.1063	*0.0376**	8.675 (3.694)	7.616 (5.786)	0.9251	0.9119	0.9393
Sum	45.41	27.08		87.09	67.92				52.05	45.70			

3B. AKR Hypothermia

Dose	**PRE VEH Males (N=5)**	**PRE VEH Females (N=5)**	**PRE VEH/Sex P value**	**Pre THC Males (N=10)**	**Pre THC Females (N=10)**	**PRE THC/Sex P value**	**PRE Male VEH/THC P value**	**PRE Female VEH/THC P value**	**Post VEH Males (N=4)**	**Post VEH Females (N=4)**	**POST VEH/Sex P value**	**Pre/Post Male VEH P value**	**Pre/Post Female VEH P value**
0	1.256 (0.698)	-0.576 (0.304)	0.1622	-0.657 (0.495)	-0.369 (0.333)	0.9999	0.2254	>0.9999	-7.735 (0.827)	-12.705 (3.478)	0.6499	*<0.0001**	*<0.0001**
1	0.648 (0.648)	-0.362 (0.431)	0.7762	-1.514 (0.334)	-0.551 (0.381)	0.9261	0.1240	>0.9999	___	___	___	___	___
3	0.172 (0.844)	-0.998 (0.323)	0.6416	-2.595 (0.441)	-0.949 (0.481)	0.5135	*0.0218**	>0.9999	3.465 (2.111)	0.220 (2.026)	0.9285	0.3504	0.9896
10	-1.022 (0.415)	-0.992 (0.465)	>0.9999	-6.507 (0.391)	-3.310 (0.793)	*0.0146**	*<0.0001**	0.3766	1.663 (2.437)	-4.445 (1.446)	0.4198	0.5671	0.5336
30	0.120 (0.798)	-1.472 (0.391)	0.2940	-7.649 (0.556)	-4.696 (0.939)	*0.0297**	*<0.0001**	0.0833	-2.560 (2.464)	-7.305 (1.527)	0.6954	0.5688	0.0758
100	-0.264 (0.710)	-1.528 (0.542)	0.5577	-14.448 (0.895)	-14.980 (1.600)	0.9963	*<0.0001**	*<0.0001**	-7.735 (0.827)	-12.705 (3.478)	0.6499	*0.0013**	*<0.0001**
130	___	___	___	___	___	___	___	____	-8.635 (0.531)	-16.290 (4.619)	0.1862	___	___
Mean	0.1517 (0.3172)	-0.9980 (0.1904)	0.1645	-5.562 (2.109)	-4.143 (2.279)	0.0554	*<0.0001**	*0.0029**	-3.590 (2.147)	-8.872 (2.511)	0.0977	0.1220	*0.0098**
Sum	0.9100	-5.928		-33.37	-24.86				-21.54	-53.23			

Supplementary Table 4 Sex-differences in acute antinociception and hypothermia in CBA mice receiving vehicle

Data represent the Means and SEM (standard error of the mean) in Percent Maximum Possible Effect (%MPE) for Tail Flick (A) and Percent Change in Body Temperature (%ΔBT) for Hypothermia (B) in CBA Mice. For the first two columns (PRE VEH), the mean and SEM for each sex is given for each dose following the administration of vehicle (VEH only) with the p value comparing sex within each dose listed to the right. For the next set of columns, the mean and SEM for mice getting THC (0-100 mg/kg; PRE THC) is listed with the P value to the right comparing sex within each dose with subsequent sets of p values comparing naïve mice getting VEH to those getting THC within each sex. Following seven days of once-daily treatment with vehicle (VEH) or 30 mg/kg THC, all mice were tested across a range of doses (0-130 mg/kg) of THC to generate post (POST) dose-response curves. The mean and SEM for each dose is listed for mice that received vehicle for 7 days prior to being assessed for the post dose-response curve of THC. P values assess sex differences in the post dose-response curve within each dose and within each sex compare differences between Pre and Post curves in VEH mice. *Significant differences are italicized; lines denote instances where values or comparisons are not applicable

4A. CBA Tail-flick

Dose	**PRE VEH Males (N=5)**	**PRE VEH Females (N=5)**	**PRE VEH/Sex P value**	**Pre THC Males (N=11)**	**Pre THC Females (N=11)**	**PRE THC/Sex P value**	**PRE Male VEH/THC P value**	**PRE Female VEH/THC P value**	**Post VEH Males (N=4)**	**Post VEH Females (N=4)**	**POST VEH/Sex P value**	**Pre/Post Male VEH P value**	**Pre/Post Female VEH P value**
0	6.520 (6.469)	6.340 (4.145)	>0.9999	-2.936 (5.261)	3.555 (7.319)	0.9766	0.8891	>0.9999	10.250 (0.550)	-32.550 (21.350)	0.3821	0.7383	0.7657
1	9.640 (2.205)	6.300 (7.039)	0.9997	7.209 (3.268)	9.727 (6.168)	0.9999	>0.9999	0.9999	___	___	___	___	___
3	19.040 (4.611)	8.000 (8.644)	0.8645	21.855 (5.519)	23.745 (7.890)	>0.9999	0.9998	0.7036	21.000 (11.800)	24.150 (8.050)	>0.9999	0.5786	0.3045
10	24.218 (4.867)	1.670 (8.778)	0.1745	21.408 (3.973)	45.771 (8.306)	*0.0406**	0.9998	*0.0019**	35.400 (15.700)	18.950 (10.550)	0.9783	0.9920	*0.0022**
30	21.120 (7.891)	7.960 (5.902)	0.7419	68.664 (9.134)	63.109 (6.690)	0.9895	*<0.0001**	*<0.0001**	60.845 (5.515)	68.175 (31.825)	0.9997	*0.0003**	*0.0032**
100	17.340 (5.445)	6.400 (13.574)	0.8693	93.664 (4.551)	92.927 (3.818)	>0.9999	*<0.0001**	*<0.0001**	74.800 (10.000)	55.600 (17.400)	0.9546	*<0.0001**	*<0.0001**
130	___	___	___	___	___	___	___	____	90.750 (9.250)	39.150 (19.750)	0.2073	___	___
Mean	16.31 (2.796)	6.112 (0.9466)	0.2551	34.98 (15.43)	39.81 (14.00)	0.3646	*0.0008**	*0.0014**	48.84 (12.93)	28.91 (14.44)	0.0825	*0.0028**	*0.0002**
Sum	97.88	36.67		209.9	238.8				293.0	173.5			

4B. CBA Hypothermia

Dose	**PRE VEH Males (N=5)**	**PRE VEH Females (N=5)**	**PRE VEH/Sex P value**	**Pre THC Males (N=11)**	**Pre THC Females (N=11)**	**PRE THC/Sex P value**	**PRE Male VEH/THC P value**	**PRE Female VEH/THC P value**	**Post VEH Males (N=4)**	**Post VEH Females (N=4)**	**POST VEH/Sex P value**	**Pre/Post Male VEH P value**	**Pre/Post Female VEH P value**
0	-1.820 (1.102)	0.440 (1.183)	0.5271	-1.500 (0.490)	0.091 (0.272)	0.4809	>0.9999	0.9998	1.575 (0.733	-0.400 (0.593)	0.7144	0.1177	0.6504
1	-1.520 (0.840)	0.180 (1.231)	0.8014	-1.109 (0.547)	0.618 (0.283)	0.3834	0.9998	0.9992	—	—	—	—	—
3	-1.280 (0.522)	-0.340 (0.969)	0.9862	-1.427 (0.565)	0.800 (0.260)	0.1323	>0.9999	0.8928	2.925 (0.125)	1.150 (0.902)	0.8014	0.4720	0.9997
10	-2.020 (0.917)	-0.880 (0.993)	0.9639	-4.018 (0.641)	-2.645 (0.762)	0.6475	0.5753	0.5292	1.025 (1.424)	-1.225 (0.634)	0.5825	*0.0321**	*0.0345**
30	-1.820 (1.386)	-0.720 (0.887)	0.9696	-7.355 (1.124)	-7.655 (0.996)	0.9998	*0.0004**	*<0.0001**	-2.300 (1.304)	-2.225 (0.545)	>0.9999	*<0.0001**	*<0.0001**
100	-2.280 (1.058)	-1.340 (0.590)	0.9862	-8.564 (0.958)	-9.436 (0.632)	0.9375	*<0.0001**	*<0.0001**	-5.500 (1.543)	-6.175 (1.573)	0.9981	*<0.0001**	*<0.0001**
130	—	—	—	—	—	—	—	—	-2.625 (1.238)	-4.200 (0.698)	0.8743	—	—
Mean	-1.79 (0.1447)	-0.4433 (0.2739)	0.3419	-3.995 (1.333)	-3.038 (1.828)	0.1197	*0.0450**	*0.0031**	-0.8167 (1.298)	-2.179 (1.083)	*0.0267**	*<0.0001**	*<0.0001**
Sum	-10.74	-2.66		-23.97	-18.23				-4.90	-13.08			
